# Targeting the Cardiovascular-Alzheimer’s Disease Axis: The Promise of Marine Bioactive Peptides

**DOI:** 10.3390/md24020056

**Published:** 2026-01-29

**Authors:** Chathuri Kaushalya Marasinghe, Kumju Youn, Chi-Tang Ho, Mira Jun

**Affiliations:** 1 Department of Food Science and Nutrition, Dong-A University, Busan 49315, Republic of Korea; chathuri93@dau.ac.kr; 2 Department of Health Sciences, The Graduate School of Dong-A University, Busan 49315, Republic of Korea; kjyoun@dau.ac.kr; 3 Center for Food & Bio Innovation, Dong-A University, Busan 49315, Republic of Korea; 4 Department of Food Science, Rutgers University, New Brunswick, NJ 08901, USA; ctho@sebs.rutgers.edu

**Keywords:** cardiovascular diseases, Alzheimer’s disease, CVD-AD axis, marine bioactive peptides, functional foods

## Abstract

Cardiovascular diseases (CVDs) and Alzheimer’s disease (AD) are among the most prevalent chronic conditions, contributing significantly to global morbidity and healthcare burdens. These diseases are increasingly recognized as interconnected through shared mechanisms such as vascular dysfunction, oxidative stress, hypertension, and systemic inflammation, collectively referred to as the CVD-AD axis. Although therapeutic strategies exist for each condition, integrated approaches targeting these common pathways remain limited. This review highlights marine-derived bioactive peptides (BAPs) as multifunctional, sustainable agents for the simultaneous prevention of CVD and AD. It summarizes recent advances in their production, purification, and characterization, with emphasis on enzymatic hydrolysis and separation techniques. Marine BAPs exhibit diverse bioactivities, antioxidant, anti-inflammatory, lipid-lowering, antihypertensive, and neuroprotective, addressing key pathological mechanisms of the CVD-AD axis. Their small size, stability, and favorable safety profile support absorption and initial bioavailability, while sustainable sourcing from underutilized marine biomass enables eco-friendly production. Despite their potential, barriers to scalable production, product standardization, and regulatory approval remain; however, incremental advances are being made toward overcoming these issues. Together with these advances, marine BAPs remain promising candidates for functional foods and nutraceuticals, providing integrated preventive strategies for age-related diseases and supporting long-term cardiovascular and cognitive health.

## 1. Introduction

The escalating global prevalence of chronic diseases, particularly cardiovascular diseases (CVD) and Alzheimer’s disease (AD), poses a growing threat to public health and healthcare sustainability. CVD, the most prevalent non-communicable disease globally, accounted for 32% of all deaths in 2021, totaling 17.9 million fatalities [1]. This burden is projected to nearly double by 2050, with a disproportionate impact in low- and middle-income countries where access to preventive care remains limited [2]. The economic consequences are equally severe, with U.S. healthcare costs expected to exceed USD 1.3 trillion annually by 2050 [3,4]. In parallel, AD, which accounts for 60 to 80% of dementia cases, currently affects over 50 million people and is projected to reach 152 million by 2050. A new case is diagnosed every three seconds, underscoring the urgent need for coordinated intervention strategies [5].

CVD encompasses a wide range of disorders that affect the heart and blood vessels, including coronary artery disease, hypertension, heart failure, and stroke. These conditions are primarily characterized by impaired blood flow, arterial stiffness, and endothelial dysfunction, often resulting from atherosclerosis and chronic inflammation. In addition to their cardiovascular consequences, CVD has been increasingly implicated in cognitive impairment due to chronic cerebral hypoperfusion and microvascular damage in the brain.

AD is a progressively deteriorating neurodegenerative disorder characterized by early memory loss and eventual multi-domain cognitive impairment. Its pathology is driven by the extracellular deposition of amyloid-beta (Aβ) plaques and the intracellular accumulation of hyperphosphorylated tau proteins in the form of neurofibrillary tangles. These pathological changes disrupt synaptic communication, trigger neuroinflammation, and lead to widespread neuronal loss. Clinical diagnosis relies on cognitive testing, neuroimaging, and cerebrospinal fluid biomarkers, such as reduced Aβ_42_ and increased total or phosphorylated tau [6].

Although traditionally considered distinct, CVD and AD are now recognized as pathophysiologically interconnected. This relationship is now referred to as the CVD-AD axis, a conceptual framework that highlights their bidirectional interactions through shared mechanisms such as vascular inflammation, oxidative stress, endothelial damage, and impaired lipid metabolism. Vascular pathology in CVD can impair cerebral perfusion and compromise the blood–brain barrier (BBB), thereby facilitating amyloid accumulation and tau phosphorylation. Conversely, AD-related neurodegeneration may dysregulate autonomic cardiovascular control, further exacerbating vascular pathology. Epidemiological studies support the idea that midlife hypertension, diabetes, and dyslipidemia significantly elevate the risk of late-life AD [1,5].

The complexity of the CVD-AD axis underscores the need for multifactorial dietary strategies for disease prevention and management. Marine ecosystems offer a largely untapped source of bioactive compounds with potential disease-modifying properties. Adapted to extreme conditions such as high salinity, temperature fluctuations, and low nutrient availability, marine organisms produce structurally diverse metabolites through unique biochemical pathways [7]. Among these, marine-derived bioactive peptides (BAPs) have attracted growing interest due to their potent biological activities, chemical stability, and favorable safety profiles. Evidence also suggests that dietary incorporation of BAPs can provide multifunctional benefits, potentially mitigating the onset or progression of both CVD and AD [8].

## 2. Exploring Marine BAPs as Promising Preventive Resource Against CVD and AD

In recent decades, bioactive natural compounds have garnered significant attention for their preventive potential [9]. Among them, “blue foods”, derived from aquatic environments such as marine algae, fish, mollusks, and other ocean-based organisms, have emerged as promising sources of pharmacological and nutraceutical agents. Given that oceans cover more than 70% of the Earth’s surface, marine environments represent a vast and largely unexplored reservoir of chemically diverse bioactive [10]. Adaptation to extreme marine conditions, such as high pressure, salinity fluctuations, low temperatures, and limited nutrients, has driven the evolution of unique metabolic pathways in marine organisms. These pathways produce structurally novel and biologically potent molecules that are rarely found in terrestrial species [7]. According to the World Register of Marine Species [11], over 368,000 accepted species have been documented, highlighting the immense chemical and biological diversity that remains underutilized.

Marine-derived compounds encompass a diverse array of bioactive molecules, including polysaccharides, polyunsaturated fatty acids (PUFAs), polyphenols, pigments, proteins, enzymes, and vitamins. Among these, marine-derived BAPs have received particular attention due to their multifaceted biological properties and relatively low toxicity [12]. These peptides, typically composed of 3–20 amino acid residues, exhibit activities that are largely dependent on their primary sequence, hydrophobicity, molecular weight, and charge distribution [8]. Low molecular weight peptides (<3000 Da) are of particular interest, as their small size facilitates transport across the intestinal epithelium, resulting in improved systemic bioavailability [13]. Compared to terrestrial peptides, marine BAPs often exhibit enhanced bioactivity, thermal and enzymatic stability, and reduced immunogenic potential. These attributes are primarily associated with their unique amino acid composition and secondary structure [14].

In the context of chronic disease prevention, particularly for CVD and AD, marine-derived BAPs have been increasingly investigated as promising modulators of shared pathological processes such as inflammation, oxidative stress, and endothelial dysfunction [15]. Although synthetic therapeutics including statins, anti-inflammatory agents, and anticoagulants are widely used in managing these conditions, their long-term use is frequently associated with adverse outcomes, including hepatic and renal toxicity, gastrointestinal disturbances, hemorrhagic risk, and polypharmacy complications [16,17]. Moreover, concerns regarding poor biodegradability and environmental accumulation have further limited their appeal in sustainable healthcare [18,19].

## 3. Biodiversity of Marine BAPs Sources: From Microbes to Vertebrates

### 3.1. Major BAP-Producing Organisms

The marine environment hosts an extraordinary diversity of life, ranging from microorganisms to vertebrates, each representing a potential source of BAPs. Microorganisms, including bacteria, fungi, and microalgae, are particularly attractive due to their rapid growth, ease of cultivation, and capacity to produce structurally diverse peptides with potent bioactivities. Macro-organisms, such as seaweeds, sponges, cnidarians, mollusks, segmented worms, arthropods, echinoderms, and chordates, contribute additional biochemical complexity, producing peptides with unique sequences, structural motifs, and multifunctional properties [20].

#### 3.1.1. Marine Microorganisms and Algae

Marine microorganisms, encompassing bacteria, fungi, and actinomycetes, are prolific sources of bioactive compounds demonstrating diverse preventive potential. Their remarkable resilience, enabling survival in extreme marine environments characterized by high salinity, intense pressure, nutrient scarcity, and low temperatures, drives the production of unique BAPs via fermentation and secondary metabolism. This adaptation yields biomolecules with significant biological activities [21,22].

Similarly, both microalgae and macroalgae (seaweed) stand out as invaluable reservoirs of protein-rich BAPs. Seaweed has attracted considerable interest within the food industry. Their rich composition of bioactive compounds, including proteins, peptides, peptide derivatives, amino acids, and other amino acid-like metabolites, positions them as key ingredients for nutraceuticals and functional foods [23]. Beyond their nutritional value, macroalgae proteins, found within cell wall structures, pigments, enzymes, and lectins, are pivotal in determining their promise as BAP sources [24]. While protein content in seaweed is species-dependent, red algae generally exhibit the highest levels, reaching up to 47% of their dry weight. The green algae follow with 9–26%, and brown algae with 3–15%. For instance, *Porphyra tenera* and *Palmaria palmata* (red algae) boast protein levels of 47% and 35% (*w*/*w*), respectively, and the green alga *Ulva pertusa* contains approximately 26% (*w*/*w*) protein [25]. It is important to note, however, that their protein composition is influenced by environmental factors such as temperature, light exposure, and water salinity, which regulate nutrient availability and nitrogen levels [26].

#### 3.1.2. Marine Invertebrates

Comprising a highly diverse and ecologically significant group, marine invertebrates thrive across a vast array of saltwater environments. This group encompasses species like sponges, corals, cnidarians, polychaetas, mollusks, crustaceans, and echinoderms [27]. Their ubiquitous distribution spans marine ecosystems from the intertidal zone to the deep ocean. Historically, coastal populations have long valued marine invertebrates for both dietary and medicinal applications, a tradition deeply rooted in cultural heritage [28]. Comprehensive chemical profiling of these organisms, including sponges, cnidarians, mollusks, and echinoderms, frequently highlights the predominance of hydrosoluble proteins. Moreover, the BAPs originating from marine invertebrates often correlate with their unique amino acid profiles and manifest a broad spectrum of pharmacological properties. Notably, glycine, arginine, glutamic acid, and aspartic acid are typically present in the highest concentrations, thereby enhancing the diverse bioactivities attributed to these organisms [29].

Additionally, shellfish, particularly crustaceans and mollusks, are recognized as a valuable source of high-quality protein, with protein content ranging between 7% and 23% (*w*/*w*) [30,31], making them an attractive candidate for BAP development. In crustaceans, both edible (muscle or meat, ~65% *w*/*w*) and inedible (heads, shells, and tails, 35–45% *w*/*w*) portions contain significant amounts of protein, further highlighting their potential as BAP sources [32]. Furthermore, recent reviews have elucidated the vast functional properties of peptides derived from marine invertebrates, underscoring their promising roles in modern preventive development [33,34].

#### 3.1.3. Marine Vertebrates

Fish represents a vital source of high-quality protein globally, serving as a staple in the human diet due to its rich composition of macronutrients and micronutrients, including essential amino acids, vitamins, minerals, and long-chain polyunsaturated fatty acids. Beyond its nutritional value, fish proteins have garnered significant attention as novel sources of BAPs with diverse physiological benefits. Fish bioactive compounds, naturally occurring in various tissues such as muscle, skin, bones, and internal organs, exhibit a broad spectrum of bioactivities, including antioxidants, anti-inflammatory, anticancer, and antimicrobial properties [35]. Fish-derived bioactive compounds encompass a wide range of molecules, including peptides, proteins, lipids, pigments, and minerals, which have been linked to improvements in cardiovascular health, cognitive function, and immune modulation [36,37]

Collectively, the marine ecosystem stands out as an exceptional source of BAPs, owing to its rich biodiversity and the remarkable adaptability of marine organisms to extreme environments. Organisms ranging from microorganisms and algae to invertebrates and vertebrates contribute to a wide array of structurally and functionally diverse peptides. These marine-derived BAPs hold great promise as sustainable resources for the development of pharmaceutical, nutraceutical, and cosmeceutical products. However, their successful application depends on effective methods for peptide preparation, purification, and characterization, which are discussed in the following section. These marine-derived BAPs hold great promises for the development of pharmaceutical, nutraceutical, and cosmeceutical products. However, their sustainable utilization depends on responsible sourcing, production efficiency, and the adoption of alternative biotechnological approaches for scale-up.

### 3.2. Major BAP-Producing Technologies

#### 3.2.1. Extraction Methods

A variety of strategies have been developed to release BAPs from marine organisms, with significant implications for their bioactivity, stability, and translational potential. These methods can be broadly categorized into conventional and emerging approaches, each offering distinct advantages and limitations [38]. A comparative overview of these techniques, outlining their principles, benefits, and limitations, is provided in Table 1.

The selection of an appropriate extraction and purification method depends on the desired characteristics of the final peptide product, including bioactivity, stability, and intended application. As summarized in Table 2, conventional enzymatic hydrolysis followed by molecular weight-based fractionation and chromatographic purification remain the most widely employed strategy for marine-derived BAP production, reflecting its broad applicability across diverse marine organisms [39,40,41,42,43]. However, comparison of the reported studies indicates that enzymatic hydrolysis alone frequently necessitates prolonged processing times and multiple downstream purification steps to isolate low molecular-weight bioactive peptides. In addition, several studies summarized in Table 2 report the use of microbial fermentation-based approaches, often coupled with chromatographic purification, which represent a biologically driven and comparatively natural processing route for peptide generation [44,45].

Notably, Table 2 highlights the increasing use of process-intensified and emerging methodologies, including microwave-assisted enzymatic hydrolysis, ultrasonic-assisted hydrolysis, and subcritical water hydrolysis, which are primarily applied to facilitate more rapid peptide release and to enrich short peptide fractions. Microwave-assisted enzymatic hydrolysis, in particular, is associated with accelerated protein hydrolysis through rapid volumetric heating and enhanced enzyme–substrate interactions, enabling efficient generation of short peptides (<1 kDa) under comparatively reduced processing durations [46]. Ultrasonic-assisted hydrolysis similarly promotes protein unfolding via cavitation effects [47], whereas subcritical water hydrolysis enables direct peptide generation without enzymatic treatment, although requiring careful control of reaction conditions [48].

While multi-step chromatographic purification remains indispensable for definitive peptide identification and structure–activity relationship analysis, the collective evidence presented in Table 2 suggests that hybrid strategies combining enzymatic hydrolysis with physical enhancement techniques are increasingly favored to reduce processing complexity and time, particularly in studies targeting functional food and nutraceutical applications. In addition, integrated approaches such as fermentation-assisted processing and simulated gastrointestinal digestion further expand peptide diversity and better reflect physiological relevance. Accordingly, Table 2 provides a comparative overview of both established and emerging extraction strategies employed for marine-derived BAPs with reported cardiovascular and neuroprotective activities.

**Table 1:** Comparison of extraction principles and methods for marine BAPs with their advantages and limitations.

Extraction	Principle	Advantages	Limitations	Refs
Enzymatic hydrolysis	Proteolytic enzymes cleave peptide bonds	Specificity, mild conditions, high bioactivity	Enzyme cost, requires optimization	[39,40]
Microbial fermentation	Proteolytic microbes secrete enzymes to break down proteins	Natural process, multifunctional bioactivity	Sensitive to pH/temp, strain selection critical	[44,45]
Chemical hydrolysis	Acidic or alkaline conditions break peptide bonds	Fast, useful for large-scale processing	Low selectivity, possible peptide degradation	[38]
Ultrasound-assisted	Ultrasonic waves disrupt protein structures	Accelerates hydrolysis, eco-friendly	Equipment cost, potential overheating	[46]
Microwave-assisted	Electromagnetic waves induce rapid protein breakdown	Energy-efficient, timesaving	May denature sensitive peptides	[46]
High hydrostatic pressure	Intense pressure alters protein conformation	Enhances enzyme accessibility	High equipment cost, limited industrial use	[47]
Subcritical water processing	Uses pressurized hot water as a solvent	Eco-friendly, improves hydrolysis efficiency	Requires specialized reactors	[48]
Pulsed electric field (PEF)	High-voltage pulses disrupt cell membranes	Improves peptide release, minimal heat	Still under development for peptides	[49]

#### 3.2.2. Purification and Identification of BAPs

Once extracted, the next critical step involves verifying the bioactivity and specificity of marine BAPs through systematic purification and characterization. Following the preparation of protein hydrolysates, assessing their bioactivity is a critical step in determining their preventive potential. This evaluation involves analyzing key physicochemical properties such as molecular weight, charge, polarity, solubility, and intermolecular interactions [50]. Upon confirming bioactivity, the next step is to fractionate the hydrolysates to isolate individual peptide fractions. Bioactive hydrolysates are subsequently subjected to fractionation using techniques such as ultrafiltration, precipitation, and chromatographic methods like high-performance liquid chromatography (HPLC) and fast protein liquid chromatography (FPLC), which enable the separation of peptides based on size, charge, or hydrophobicity. The resulting fractions are then characterized through mass spectrometry and protein sequencing, which provide detailed information on amino acid composition, structural features, and potential functional motifs [51,52].

These purification strategies have been adapted based on the biochemical complexity of different marine organisms (Table 2). For example, peptide purification from microalgae such as *Spirulina maxima* and *Chlorella pyrenoidosa* typically involves enzymatic hydrolysis followed by ultrafiltration (e.g., 1–10 kDa cutoffs), RP-HPLC, and LC-MS/MS [53,54,55]. In macroalgae such as *Gracilariopsis chorda* or *Palmaria palmata*, peptide purification begins with papain or pepsin hydrolysis and proceeds through sequential chromatographic steps. These include gel filtration using Sephadex G-25, C18 RP-HPLC, and, in some cases, solid-phase peptide synthesis (SPPS) for structural verification [56,57]. Marine invertebrates such as *Stichopus japonicus*, *Apostichopus japonicus*, and *Marphysa sanguinea* require species-specific workflows, often incorporating ceramic membrane filtration, DEAE columns, or nano-ESI Q-TOF-MS systems for peptide profiling [58,59,60]. Fish-derived peptides are also purified using species-specific workflows that involve enzymatic hydrolysis, membrane ultrafiltration, and RP-HPLC coupled with LC-MS/MS for structural characterization [61,62]. This taxon-dependent variability highlights the importance of optimizing the choice of enzymes, pH levels, and downstream purification parameters for each source material to achieve functionally valid and structurally defined peptides.

To accelerate discovery and functional assessment of novel peptides, advanced technologies are increasingly integrated into BAP research. In silico approaches, such as molecular docking and quantitative structure–activity relationship (QSAR) modeling integrate bioinformatics with experimental validation to predict peptide structures, activities, and targets, thereby accelerating discovery [63]. Phage display technology further enhances peptide screening by enabling rapid identification of sequences with high target specificity. Additionally, bead-based global proteomic screening (Bead-GPS) combines microarray platforms with label-free mass spectrometry, facilitating high-throughput identification of BAPs from complex mixtures [64]. For example, a study on abalone meat identified 1937 peptide sequences through separation and peptidomic analysis, and virtual screening coupled with molecular docking highlighted 14 peptides with ACE inhibitory activity [65]. Similarly, the study computationally analyzed RuBisCO proteins from commonly consumed seaweed to predict bioactive peptides, many of which were di- and tripeptides with potential DPP-IV, ACE inhibitory, antioxidant, anti-inflammatory, and anticancer activities [66].

Ultimately, these purification and identification strategies not only ensure the structural and functional validation of marine BAPs but also enhance their bioavailability, safety, and translational potential for diet-based prevention of CVD and AD. By integrating traditional and emerging approaches, researchers can produce peptides suitable for functional foods, nutraceuticals, and dietary interventions, aligning with the broader goals of food science and nutrition in preventing chronic, diet-related diseases.

**Table 2:** Comprehensive strategies for extracting and purifying BAPs from marine sources.

	Peptide	Species	Extraction	Purification and Identification	Basic Characteristics	Ref.
Micro organisms (Fungus)	Cyclic tetrapeptide, Aspochracin-type cyclic tripeptide, Sclerotiotide L, Diketopiperazine dimer 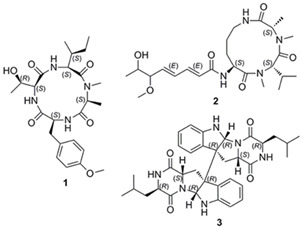	*Aspergillus violaceofuscus*	Microbial fermentation, Ethyl acetate extraction	Vacuum liquid chromatography, MPLC, RP-HPLC	Microbial fermentation (80 g rice, 120 mL H_2_O, 0.3% peptone), Vacuum chromatography (silica gel 200–300 mesh, CH_2_Cl_2_/MeOH gradient 500:1–0:1), Sephadex LH-20 (MeOH), MPLC on ODS (10–100% MeCN/H_2_O gradient), RP-HPLC (55% MeOH, 2.0 mL/min)	[67]
Algae	* Tyr-Ile-Gly-Asn-Asn-Pro-Ala-Lys-Gly-Gly-Leu-Phe (MW: <3 kDa) 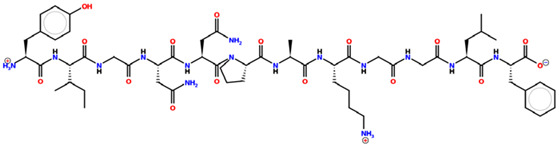 * Ile-Gly-Asn-Asn-Pro-Ala-Lys-Gly-Gly-Leu-Phe (MW: <3 kDa) 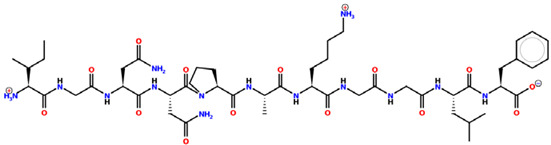	*Laminaria digitata*	Chemical synthesis	MS spectroscopy	MWCO filtration, Peptide clean-up (Phoenix kit), LC-MS/MS (nanoESI qQTOF, 6600 plus TripleTOF), RP-HPLC-MS (purity verification)	[68]
	* Leu-Asp-Ala-Val-Asn-Arg (MW: 686 Da) 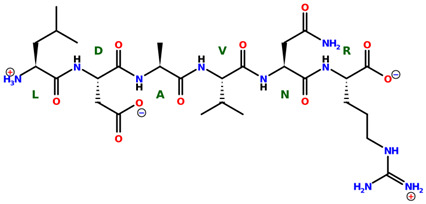	*Spirulina maxima*	Enzymatic hydrolysis	Ultrafiltration, Anion-exchange chromatography, Gel filtration, RP-HPLC, LC-MS/MS	Trypsin (pH 8, 37 °C), α-Chymotrypsin (pH 8, 37 °C), Pepsin (pH 2, 37 °C), Ultrafiltration (10, 5, 3 kDa membranes), Anion-exchange chromatography (DEAE FF, 2 mL/min), Gel filtration (Superdex Peptide 10/300 GL), RP-HPLC (C18, 1 mL/min), LC-MS/MS (Q-TOF, ESI source)	[53]
	* Val-Glu-Cys-Tyr-Gly-Pro-Asn-Arg-Pro-Gln-Phe (MW: 1309 Da) 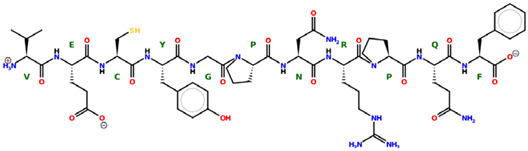	*Chlorella pyrenoidosa*	Enzymatic hydrolysis	Ultrafiltration, Gel filtration, RP-HPLC, LC-MS/MS	Pepsin (pH 2, 37 °C), Flavourzyme (pH 7, 50 °C), Alcalase (conditions per manufacturer), Papain (conditions per manufacturer), Sephacryl S-100 column (2.6 × 70 cm), Q-Sepharose Fast Flow column (2.6 × 40 cm,), Superdex Peptide HR 10/30 column (0.5 mL/min), RP-HPLC (C18, gradient not specified), Agilent 6510 Q-TOF MS	[54]
	MW < 1kDa, 1–3 kDa, 3–10 kDa, >10 kDa peptides		Enzymatic hydrolysis	Ultrafiltration	Pepsin (1 mg/mL), centrifuged (1.2× *g* rpm, 10 min), Ultrafiltration (Molecular weight cutoff: <1 kDa, 1–3 kDa, 3–10 kDa, >10 kDa)	[55]
	* Ile-Ile-Ala-Val-Glu-Ala-Gly-Cys (MW: 774.92 Da) 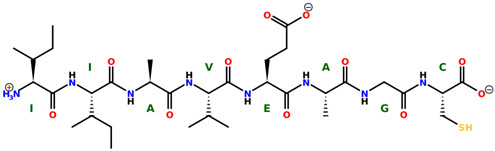	*Isochrysis zhanjiangensis*	Enzymatic hydrolysis	RP-HPLC, LC-MS/MS	Chymotrypsin (pH 7.8, 37 °C), Trypsin (pH 7.5, 37 °C), Pepsin (pH 2, 37 °C), RP-HPLC (Synchropak RPP-100, 4.6 × 250 mm, 60 mL/h), LC-MS/MS (ESI-QTOF)	[69]
	* Ile-Asp-His-Tyr (MW: 546.2 Da) 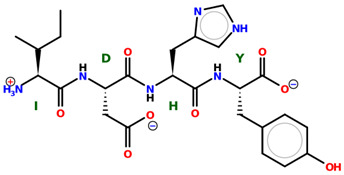	*Gracilariopsis chorda*	Enzymatic hydrolysis	Ultrafiltration, Gel filtration, RP-HPLC, LC-MS/MS	Pepsin (pH 2, 37 °C), Papain (pH 6, 37 °C), Flavourzyme (pH 7, 50 °C), Kojizyme (pH 6, 40 °C), Protamex (pH 6, 40 °C) Gel filtration: Sephadex G-25 column (2.5 × 75 cm, 1.5 mL/min) RP-HPLC: C18 ODS, 0–30% ACN, 0.8 mL/min	[56]
	* Ile-Arg-Leu-Ile-Ile-Val-Leu-Met-Pro-Ile-Leu-Met-Ala (MW: 1494.93) 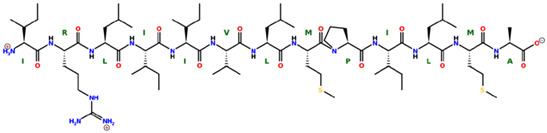	*Palmaria palmata*	Enzymatic hydrolysis	RP-HPLC, LC-MS/MS, MW-SPPS	Papain (pH 6, 60 °C, 24 h), RP-HPLC (C18, 100 × 21.2 mm, 1.0 mL/min), LC-MS/MS (nano-UPLC BEH130 C18, 100 × 0.1 mm, 250 nL/min), MW-SPPS (Liberty CEM synthesizer, H-Ala/Ile-HMPB-ChemMatrix resin), RP-HPLC (C18), MALDI-TOF MS, Lyophilization (Genevac HT 4X)	[57,70]
	<3 kDa, 3–10 kDa, >10 kDa peptides	*Ulva prolifera*	Enzymatic hydrolysis	Sephadex G-100 gel filtration, Ultrafiltration, Nano-LC-MS/MS	Neutral protease (pH 7.4, 47 °C), Ultrafiltration (<3 kDa, 3–10 kDa, >10 kDa), Sephadex G-100 column (2.5 × 70 cm, 1.0 mL/min), Nano-LC-MS/MS (online nano-flow)	[71]
	* Glu-Thr-Thr (MW: 998.02 Da) 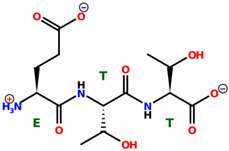	*Isochrysis zhanjiangensis*	Gastrointestinal digestion		Simulated GI digestion: Pepsin (pH 2, 37 °C, 2.5 h), Trypsin + Chymotrypsin (1:100 *w*/*w*, 37 °C, 2.5 h)	[72]
Invertebrates (Echinoderm)	* Phe-Tyr-Asp-Trp-Pro-Lys (MW: 854.4 Da) 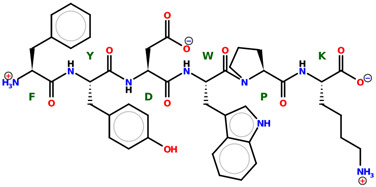	*Stichopus japonicas*	Chemically synthesized			[59]
	* Asp-Asp-Gln-Ile-His-Ile-Phe (MW: 886.4 Da) 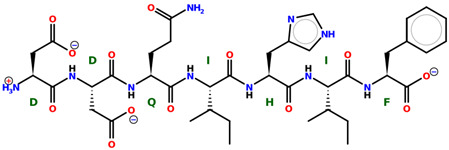 * His-Asp-Trp-Trp-Lys-Glu-Arg (MW: 1055.5 Da) 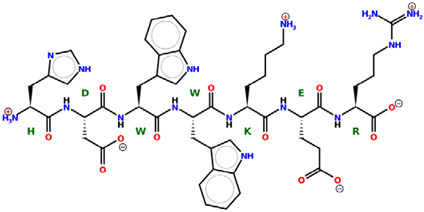 * Thr-His-Asp-Trp-Trp-Lys-Glu-Arg (MW: 1156.5 Da) 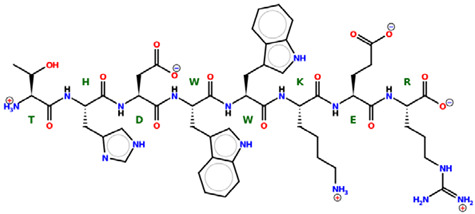	*Apostichopus japonicus*	Vacuum freeze-drying, Enzymatic hydrolysis	Ceramic membrane filtration, Ultrafiltration, Targeted affinity purification, RP-HPLC, LC-MS/MS	Pepsin (pH 2, 37 °C), Papain (pH 6, 37 °C), Flavourzyme (pH 7, 50 °C), Kojizyme (pH 6, 40 °C), Protamex (pH 6, 40 °C), Cyanogen bromide-activated agarose 4B column (pH 4.0–8.0), Sephadex G-15 column (3.6 × 150 cm, 1.0 mL/min), RP-HPLC (C18, 0.5 mL/min), LC-MS/MS (Q-Exactive, m/z 200–2000)	[58]
	* Met-Glu-Gly-Ala-Gln-Glu-Ala-Gln-Gly-Asp (MW: 1034.4 Da) 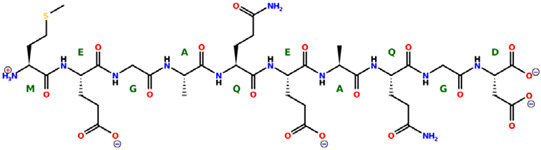	*Acaudina molpadioidea*	Enzymatic hydrolysis and Solid-phase peptide synthesis	Ultrafiltration Gel filtration, RP-HPLC, Mass spectrometry, HPLC, ODS column.	Alcalase (pH 7.5, 55 °C), Ultrafiltration (2 kDa cutoff), Sephadex G-25 column (1.6 × 30 cm, 0.6 mL/min), SP Sephadex C-25 column (2.6 × 30 cm, 0.6 mL/min), RP-HPLC (Zorbax C18, 0.8 mL/min), nanoESI-MS/MS, Solid-phase peptide synthesis	[73]
	* Phe-Leu-Ala-Pro (MW < 1 kDa) 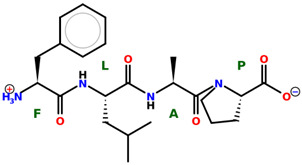		Microwave-assisted enzymatic hydrolysis	Ultrafiltration, Gel filtration, RP-HPLC	Papain (pH 6.5, 37 °C), Pepsin (pH 2, 37 °C), Trypsin (pH 8.5, 45 °C), Ultrafiltration (<1 kDa, 1–5 kDa, >5 kDa), Sephadex G-15 column, RP-HPLC (0–75% ACN, 45 min)	[74]
	* Gly-Val-Ser-Gly-Leu-His-Ile-Asp (MW: 797 Da) 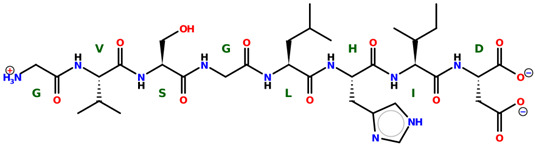	*Actinopyga lecanora*	Enzymatic hydrolysis	Ultrafiltration, SDS-PAGE, RP-HPLC, UHPLC-Q-TOF-MS/MS, Peptide synthesis	Alcalase hydrolysis (pH 7.9, 55 °C), Ultrafiltration (10, 5, 2 kDa MWCO), SDS-PAGE (15% acrylamide), Sephadex G-25 column (2.5 × 75 cm, 1 mL/min), RP-HPLC (C18, 4 mL/min), UHPLC-Q-TOF-MS/MS (positive ESI)	[75]
	Asn-Asp-Glu-Glu-Leu-Asn-Lys (MW: 860.4) 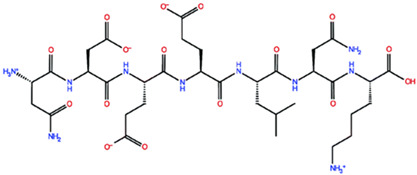	*Stichopus japonicus*	Commercial peptide		98% purity	[76]
Invertebrates (Annelida)	* Asn-Cys-Trp-Pro-Phe-Gln-Gly-Val-Pro-Leu-Gly-Phe-Gln-Ala-Pro-Pro (MW: 1757.86 Da) 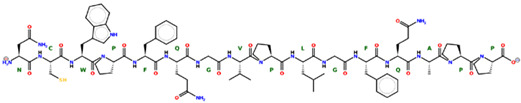	*Marphysa sanguinea*	Acetic acid extraction,	Sep-Pak C18 concentration, RP-HPLC, UPLC-Q-TOF MS/MS	Acetic acid extraction (1%, 100 °C, 5 min), Sep-Pak C18 cartridge, RP-HPLC (Delta Pak C18, 1 mL/min), UPLC-Q-TOF MS/MS (C18, 0.1 mL/min)	[60]
Invertebrates (Mollusk)	Leu-Leu-Arg-Leu-Thr-Asp-Leu (MW: 842.5 Da) 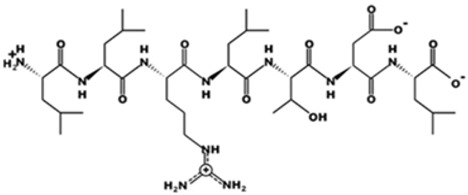 Gly-Tyr-Ala-Leu-Pro-Cys-Asp-Cys-Leu (MW: 953.4 Da) 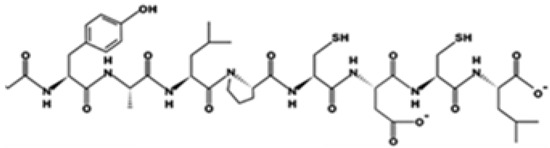 Ala-Trp-Leu-Asn-His (MW: 639.3 Da) 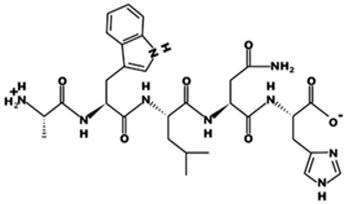 Pro-His-Asp-Leu (MW: 480.2 Da) 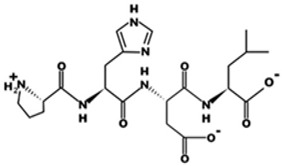	*Scapharca subcrenata*	Chemically synthesized		Synthesized peptides with >96% purity confirmed by LC-MS/MS	[77,78]
	Pro-Ile-Ile-Ser-Val-Tyr-Trp-Lys (MW: 1005.2 Da) 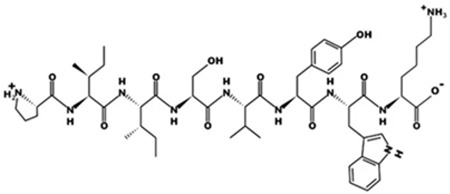 Phe-Ser-Val-Val-Pro-Ser-Pro-Lys (MW: 860.05 Da) 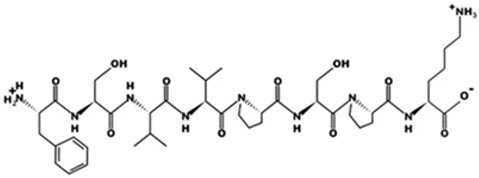	*Mytilus edulis*	Chemically synthesized		Purity >96% (LC-MS/MS)	[79]
	Pro-Ile-Ile-Ser-Val-Tyr-Trp-Lys (MW: 1004.6 Da), Phe-Ser-Val-Val-Pro-Ser-Pro-Lys (MW: 859.5 Da)		Chemically synthesized (Peptron Inc. Daejeon, Korea)	purity > 96% by LC-MS/MS	Synthesized peptides with >96% purity confirmed by LC-MS/MS	[80]
	* Glu-Pro-Thr-Phe (MW: 493 Da) 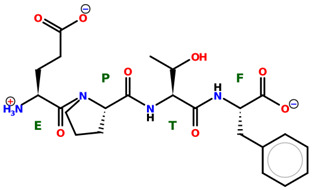 * Phe-Thr-Val-Asn (MW: 480 Da) 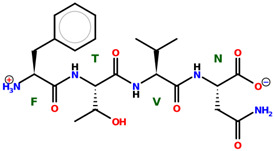		Enzymatic hydrolysis	Gel filtration, RP-HPLC, LC-MS/MS	α-Chymotrypsin (pH 8, 37 °C), Sephadex G-25 column (3.0 × 90 cm, 1.0 mL/min), RP-HPLC (C18 column, 2.0 mL/min, ACN gradient), LC-MS/MS (Q-TOF, ESI source)	[81]
	* Ile-Lys (MW: 259.33 Da) 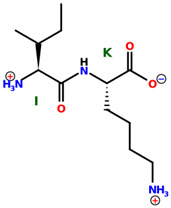 * Tyr-Glu-Gly-Asp-Pro (MW: 579.56 Da) 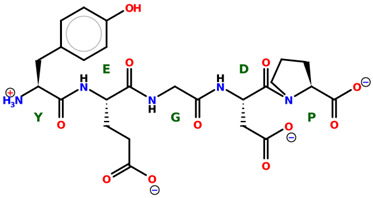 * Trp-Phe(MW: 351.41 Da) 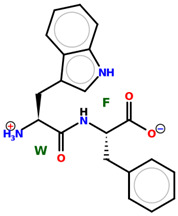 * Ser-Trp-Ile-Ser-Ser (MW: 578.62Da) 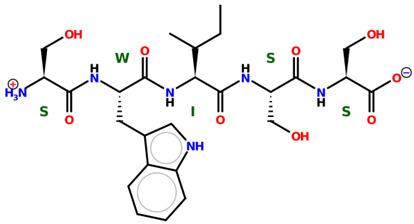		Enzymatic hydrolysis	Ultrafiltration, Ion exchange chromatography, Gel filtration, RP-HPLC	Pepsin (pH 1.5, 37 °C), Trypsin (pH 7.0, 37 °C), Ultrafiltration membranes (1, 3.5, 5, 10 kDa), QFF anion exchange column (3.8 × 150 cm, 2.5 mL/min), Sephadex G-15 column (3.6 × 150 cm, 1.0 mL/min), RP-HPLC (Zorbax C18, 4.6 × 250 mm, 5 µm, 10–60% ACN gradient)	[82]
	* Phe-Gly-His-Pro-Tyr (MW: 620 Da) 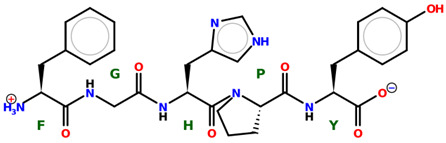		Spontaneous fermentation		25% NaCl (*w*/*w*), 20 °C, 12 months	[83]
	Peptide fraction (<1 kDa)	*Mytilus coruscus*	Enzymatic hydrolysis	Ultrafiltration	Trypsin (pH 8.0, 37 °C), Pepsin (pH 2.0, 37 °C), Papain (pH 6.0, 60 °C), Alcalase (pH 10.0, 45 °C), Neutrase (pH 7.0, 45 °C), Cogent μScale TFF ultrafiltration system, Spectra/Por dialysis tubing (MW cutoff 150 Da)	[84]
	Hydrolyzed collagen	*L. smithi* + black jelly mushroom extract	Enzymatic hydrolysis	Gel filtration chromatography, UV detection	Papain (pH 7, 40 °C, 150 min), Alcalase (pH 8, 50 °C, 120 min), Sephadex G-25 column (size not specified, absorbance at 280 nm), MW markers for molecular weight estimation	[85]
	* Gly-Val-Gly-Ser-Pro-Tyr (MW: 578.7 Da) 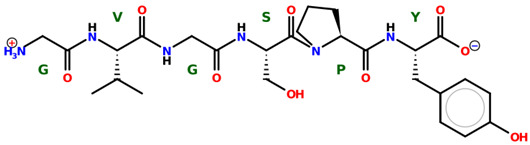	*Pinctada fucata*	Enzymatic hydrolysis	Gel filtration chromatography, RP-HPLC	Nucleicin (pH 7.0, 50 °C), Orientase 22BF (pH 9.2, 60 °C), Sephadex G-25 column (2.2 × 70 cm, 0.8 mL/min), RP-HPLC (COSMOSIL 5C18-AR-II, 0.8 mL/min)	[86]
	* Ala-Gly-Phe-Ala-Gly-Asp-Asp-Ala-Pro-Arg (MW: 975.4 Da) 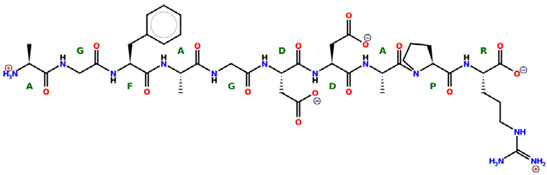 * Cys-Asp-Val-Asp-Ile-Arg (MW: 719.3 Da) 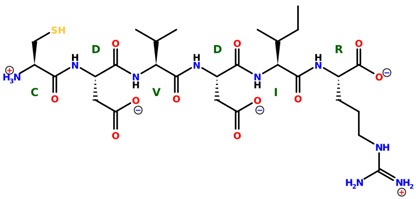	*Chlamys farreri*	Microorganism fermentation	Ultrafiltration, RP-HPLC, LC-MS/MS	*Bacillus natto* (optimized: 38.4 h, 39.9 °C, 6.0% inoculum), fermented *Chlamys farreri* skirt, Ultrafiltration: 10 and 3 kDa membranes (Pellicon XL, Millipore), fractions: <3, 3–10, and >10 kDa, Gel filtration: Sephadex G-15 column (1.6 × 40 cm, 3 mL/min), RP-HPLC: SunFire™ Prep C18 OBD™ (19 × 150 mm, 10 µm), LC-MS/MS (Mascot 2.2)	[87]
	* Asp-Leu-Thr-Asp-Tyr (MW: 625.3 Da) 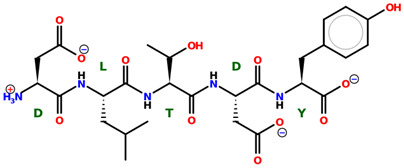	*Crassostrea gigas*	Protease hydrolysis	RP-HPLC, Amino acid sequence	Trypsin hydrolysis (0.5%, pH 7, 25 °C, 24 h), RP-HPLC (ODS-AM120-S50, 2 × 50 cm, 5 mL/min), JAI-ODS-S343-15 column (2.5 × 25 cm), FinePak-SiL-C18-5 column (0.46 × 25 cm)	[88]
	GPH-IV-P2 (MW ≈ 5 kDa)	*Perna canaliculus*	Enzymatic hydrolysis	Gel-filtration, RP-HPLC, LC-MS/MS	Mussel flesh boiled (10 min), homogenized (1500 rpm, 5 min), protein extracted, fractionated, freeze-dried	[89]
Invertebrates (Arthropoda)	* Asn-Gly-Val-Ala-Ala (MW: 431 Da) 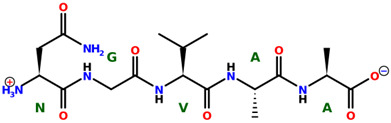	*Oratosquilla woodmasoni*	Enzymatic hydrolysis	Ultrafiltration, Ion exchange chromatography, Gel filtration chromatography	Thermolysin (70 °C, pH 8) Pepsin (37 °C, pH 2), Ultrafiltration (Amicon 3 & 10 kDa MWCO), DEAE anion exchange chromatography, Sephadex G-25 gel filtration	[90]
Invertebrates (Tunicate)	* Leu-Trp-His-Thr-His (MW: 692.2 Da) 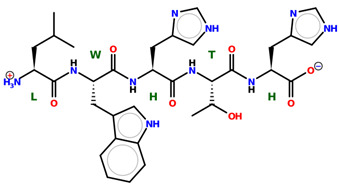	*Styela clava*	Enzymatic hydrolysis	Gel filtration, RP-HPLC, QTOF ESI-MS	Pepsin (pH 2, 37 °C), Papain (pH 6, 37 °C), Flavourzyme (pH 7, 50 °C), Kojizyme (pH 6, 40 °C), Protamex (pH 6, 40 °C), Sephadex G-25 column (2.5 × 75 cm, 1.5 mL/min), RP-HPLC (C18 ODS, 0–30% ACN, 0.8 mL/min)	[91]
Vertebrates (Fish)	Gly-Thr-Glu-Asp-Glu-Leu-Asp-Lys (MW: 906.4 Da) 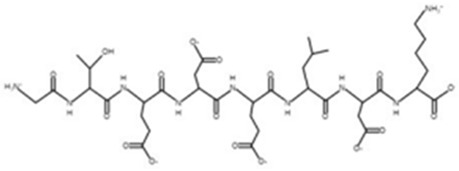	*Hippocampus trimaculatus*	Enzymatic hydrolysis	Ion exchange chromatography, RP-HPLC	Trypsin, a-chymotrypsin, papain, and pronase E), Enzyme inactivation (100 °C, 10 min), Lyophilization (−80 °C storage) Ion exchange chromatography (FPLC, DEAE FF, 4 mL/min,), RP-HPLC (Nucleosil C18, 10 × 250 mm)	[92]
	Marine collagen peptides (MCPs)	*Chum salmon* skin	Enzymatic hydrolysis	HPLC, MALDI-TOF MS	Alcalase (pH 8, 40 °C, 3 h), Protamex (pH 8, 40 °C, 3 h), HPLC (Waters Corporation), MALDI-TOF MS (LDI-1700), Amino acid analyzer (H835-50)	[93]
	* His-Gly-Ser-His (MW: 436.43 Da) 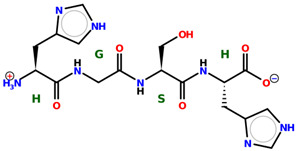 * Lys-Gly-Pro-Ser-Trp (MW: 573.65 Da) 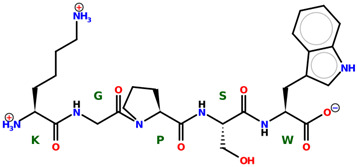	*Hippocampus abdominalis*	Enzymatic hydrolysis	Ultrafiltration, Gel filtration, RP-HPLC, LC-MS/MS	Enzymatic hydrolysis: Pepsin (pH 2, 37 °C), Papain (pH 6, 37 °C), Flavourzyme (pH 7, 50 °C), Kojizyme (pH 6, 40 °C), Protamex (pH 6, 40 °C), Gel filtration: Sephadex G-25 column (2.5 × 75 cm, 1.5 mL/min), RP-HPLC: C18 ODS, 0–30% ACN, 0.8 mL/min	[94]
	* Ser-Pro (MW: 202.3 Da) 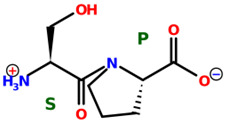 * Val-Asp-Arg-Tyr-Phe (MW: 698.9 Da) 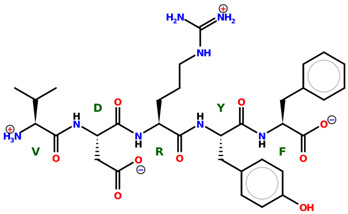	*Katsuwonus pelamis*	Enzymatic hydrolysis	Ultrafiltration, Gel filtration, RP-HPLC, LC-MS/MS	Pepsin (pH 2, 37 °C), Papain (pH 6, 37 °C), Flavourzyme (pH 7, 50 °C), Kojizyme (pH 6, 40 °C), Protamex (pH 6, 40 °C). Gel filtration: Sephadex G-25 column (2.5 × 75 cm, 1.5 mL/min). RP-HPLC: C18 ODS column, 0.8 mL/min.	[95]
	* Pro-Pro-Leu-Leu-Phe-Ala-Ala-Leu (MW: 841.05 Da) 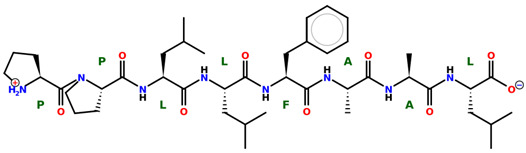	*Takifugu flavidus*	Enzymatic hydrolysis	Ultrafiltration, Gel filtration, RP-HPLC (semi-prep), RP-HPLC (analytical), LC-MS/MS	Alcalase (pH 8.0, 55 °C), Pepsin (pH 2.0, 37 °C), Neutral protease (pH 7.0, 55 °C), Ultrafiltration: MWCO membranes (1, 3, 10, 30, 50 kDa), Gel filtration: Sephadex G-15 column (0.5 × 100 cm, 5 mL/min), RP-HPLC (semi-prep): ODS-BP column (50 × 400 mm, 1 mL/min), RP-HPLC (analytical): C18 column (4.6 × 250 mm, 1 mL/min)	[61]
	* Asp-Pro-Ala-Leu-Ala-Thr-Glu-Pro-Asp-Pro-Met-Pro-Phe (MW: 1382 Da) 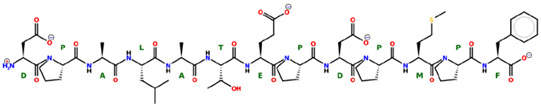	*Oreochromis niloticus*	Enzymatic hydrolysis	Ion exchange chromatography, HPLC	Alcalase, Pronase E, pepsin, and trypsin, Ion Exchange Chromatography (2 mL/min flow rate), RP-HPLC (2 mL/min flow rate)	[96]
	* Phe-Ala-Gly-Pro-Pro-Gly-Gly-Asp-Gly-Gln-Pro-Gly-Ala-Lys (MW: 1255.33 Da) 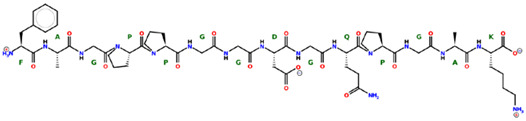 * Ile-Ala–Gly-Pro-Ala-Gly-Pro-Arg-Gly-Pro-Ser-Gly-Pro-Ala (MW: 1204.33 Da) 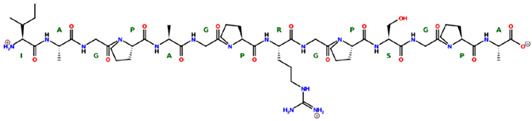	*Salmo salar*	Alkaline treatment, Gelatin extraction	Enzymatic hydrolysis, Enzyme inactivation, Ultrafiltration, RP-HPLC, nano-LC-MS/MS, HPLC analysis	Alkaline protease (pH 7.5, 55 °C), Ultrafiltration membrane (1 kDa), YMC ODS-A C18 column (1.0 cm × 10 cm, 1 mL/min), nano-LC-MS/MS (Hypersil Gold C18, 150 μm × 15 cm, 600 nL/min), SB-C18 column (4.6 mm × 250 mm, 1 mL/min)	[97]
	SWP-I (MW: 4976 Da), SWP-II (MW: 1960 Da)	*Gadus morhua*	Alkaline treatment, Enzymatic hydrolysis	Gel filtration, chromatography, UV-Vis spectroscopy, FT-IR spectroscopy	Protamex (pH 7.5, 55 °C), Heat inactivation (100 °C, 5 min), Sephadex G-15 column (2.6 × 60 cm, 1.5 mL/min), RP-HPLC (C18, 1.0 mL/min), GPC (SRT SEC-300 column, 0.5 mL/min)	[98]
	* His-Asn-Leu-Gly-Leu-Leu-His-Gly-Asp-Met-Leu (MW: 1105.52 Da) 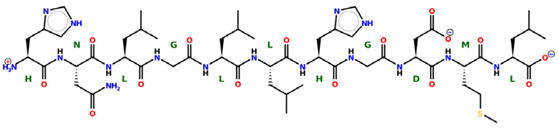 * Asp-Ala-Pro-Ser-Met-Asn-Asp (MW: 748.25 Da) 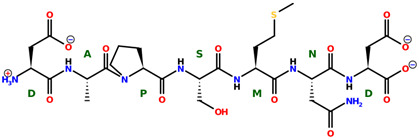	*Piaractus brachypomus*	Fermentative hydrolysis	Ultrafiltration, Gel filtration, RP-HPLC, LC-MS/MS	*Bacillus* fermentation (37 °C, 50 rpm), ultrafiltration (3 kDa MWCO), Sephadex G-25 column (2.6 × 60 cm, 0.5 mL/min), RP-HPLC (C18, 1.0 mL/min), LC-MS/MS (positive ion mode, 100–3000 *m*/*z*)	[29]
	* Asn-His-Arg-Tyr-Asp-Arg (MW: 856 Da) 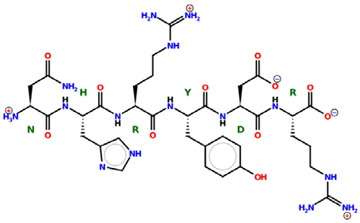 * Gly-Asn-Arg-Gly-Phe-Ala-Cys-Arg-His-Ala (MW: 1101.5 Da) 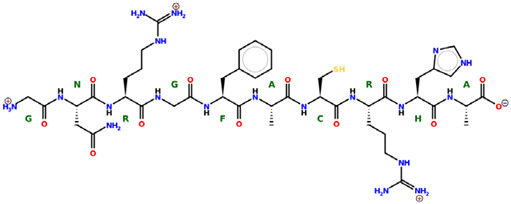	*Magalaspis cordyla*, *Otolithes ruber*	In vitro digestion	Ion exchange chromatography, Gel filtration, ESI-MS/MS	Pepsin (pH 2.5, 37 °C, 2 h), Trypsin + α-chymotrypsin (pH 8, 37 °C, 2.5 h), Ion exchange chromatography (DEAE column, 1 mL/min), Gel filtration (Sephadex G-25 column, 2.5 × 75 cm, 1 mL/min), ESI-MS/MS (FIA 3200 QTRAP, positive ion mode)	[99]
	* Lys-Ala-Pro-Asp-Pro-Gly-Pro-Gly-Pro-Met (MW: 966.1 Da) 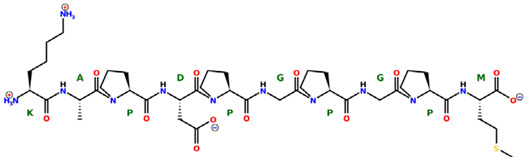	*Oreochromis niloticus*	Enzymatic hydrolysis	Ultrafiltration, RP-HPLC, LC-MS/MS, Solid-phase peptide synthesis (SPPS)	Alkaline protease (pH 10, 51 °C, 9 h), Ultrafiltration membranes (5 kDa, 3.5 kDa, 1 kDa MWCO), PREP-ODS column (20 × 250 mm, 7 mL/min), LC-MS/MS (RP C18 column, 600 nL/min)	[62]
	Low MW peptides (MW < 600 Da)	*Bigeye tuna*	Subcritical water hydrolysis		150–300 °C, 50–100 bar, 5 min	[100]
	* Gly-Ile-Ile-Gly-Pro-Ser-Gly-Ser-Pro (MW: 784 Da) 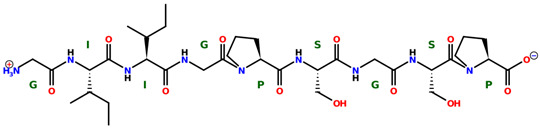 * Ile-Gly-Thr-Gly-Ile-Pro-Gly-Ile-Trp (MW: 913 Da) 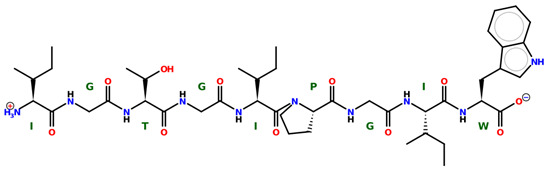 * Gln-Ile-Gly-Phe-Ile-Trp (MW: 763 Da) 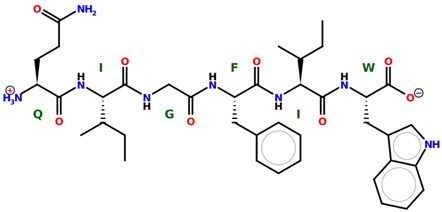	*Hippocampus abdominalis*	Enzymatic hydrolysis	Ultrafiltration, Gel filtration, RP-HPLC, LC-MS/MS	Alcalase (0.01% *w*/*w*, 24 h, 50 °C, pH 8), Ultrafiltration (5, 10 kDa), Gel filtration, RP-HPLC (T3 column)	[101]
	* Leu-Leu-Asp-Phe (MW: 506.3 Da) 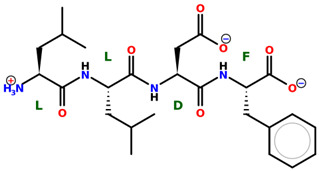	yellowfin tuna	Ultrasonic-assisted enzymatic hydrolysis	RP-HPLC, LC-MS/MS	Trypsin (pH 8.1, 37 °C), Neutral enzyme (pH 7, 50 °C), Papain (pH 6.5, 55 °C), Pepsin (pH 3, 37 °C), Alkaline enzyme (pH 9, 50 °C), 1:25 (*w*/*v*), Ultrasonication (200 W, 55 °C, 25 kHz)	[42]
	* Ser-Leu-Ala-Phe-Val-AspAsp-Val-Leu-Asn (MW: 1091.5 Da) 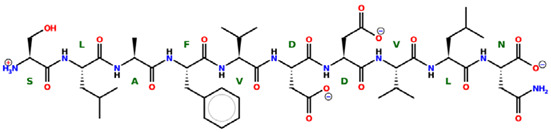	*Merluccius productus*	Enzymatic hydrolysis	Sephadex G-25 gel filtration, Preparative RP-HPLC, Analytical RP-HPLC, MS/MS	Umamizyme K hydrolysis (pH 6.6–6.9, 50 °C, 1 h), Sephadex G-25 gel filtration (25 × 750 mm), Preparative RP-HPLC (Grom-sil 120 ODS-5ST, 10 × 250 mm), Analytical RP-HPLC (C18, 4.6 × 250 mm) MS/MS (Q-TOF, nano-ESI, Capcell Pak C18 UG120 V, MeOH/H_2_O 1:1, [M+2H]^2+^ ion selection)	[102]

* Structures drawn using PepDraw software (version 4.0, Bearing Forward, LLC: USA, 2026. Available online: https://pepdraw.com, accessed on 19 January 2026).

## 4. Shared Biological Processes Linking Cardiovascular (CVD) and Alzheimer’s Diseases (AD)

### 4.1. Molecular Mechanism of CVD Progression

Oxidative stress-induced endothelial dysfunction is the earliest detectable event in the pathophysiology of CVD. Endothelial cells maintain vascular homeostasis through nitric oxide (NO) production and anti-inflammatory signaling [103]. However, chronic exposure to cardiovascular risk factors, such as hypertension, hyperglycemia, and dyslipidemia, impair these cells [104]. This dysfunction is primarily mediated by overproduction of reactive oxygen species (ROS) generated from enzymatic sources including NADPH oxidases (e.g., NOX2/NOX4) and dysfunctional mitochondria. The imbalance between ROS and antioxidant systems (e.g., superoxide dismutase, glutathione peroxidase, metallothioneins) leads to endothelial nitric oxide synthase (eNOS) uncoupling and reduced NO bioavailability [105]. Simultaneously, oxidative modifications of low-density lipoprotein (LDL) particles occur in the subendothelial space, producing oxidized LDL (oxLDL). These modified LDL particles initiate endothelial activation and subsequent immune responses, driving monocyte infiltration and foam cell formation in early atherogenesis [106].

When persistent, this activation progresses to endothelial dysfunction, which is often driven by continued oxidative stress and lipoprotein modification. This sustained dysfunction further promotes the expression of adhesion molecules such as vascular cell adhesion molecule-1 (VCAM-1), intercellular adhesion molecule-1 (ICAM-1), and E-selectin, thereby facilitating the adhesion of circulating monocytes and their transendothelial migration into the intimal layer [107,108]. Once within the vessel wall, these monocytes differentiate into macrophages, internalize oxLDL through scavenger receptors such as SR-A1, CD36, and LOX-1, and subsequently form foam cells [109]. These lipid-laden cells constitute the foundation of early atheromatous plaques. In parallel, vascular smooth muscle cells (VSMCs) migrate from the media, proliferate, and synthesize extracellular matrix components. Under inflammatory conditions, VSMCs can transdifferentiate into VSMC-derived foam cells, contributing to plaque progression [108]. Simultaneously, macrophages within the plaque become activated, amplifying local inflammation and promoting plaque progression [110].

These activated macrophages release proinflammatory cytokines, notably TNF-α, IL-1β, and IL-6, through NF-κB and NLRP3 inflammasome pathways, thereby sustaining a self-amplifying cycle of inflammation [111]. Additionally, VSMCs respond to inflammatory signals by migrating into the intima and undergoing a phenotypic switch toward a synthetic, pro-fibrotic state, further contributing to lesion complexity [104]. Foam cells, extracellular lipid deposits, and proliferating VSMCs interact to drive early plaque expansion. As the lesion enlarges, it forms a necrotic core encased by a fibrous cap composed of collagen and elastin synthesized by VSMCs. Persistent lipid deposition and VSMC proliferation further drive the progression and structural complexity of the plaque [111]. Moreover, macrophages within the lesion have exhibited impaired migratory capacity, prevented resolution of inflammation and promoted the transition toward more complex and unstable atherosclerotic plaques.

In advanced stages of CVD, fibrotic remodeling of plaques is largely mediated by TGF-β and connective tissue growth factor (CTGF). This remodeling process, coupled with oxidative and inflammatory stress, increases plaque vulnerability, potentially leading to plaque rupture and subsequent thrombotic events such as myocardial infarction or stroke [112]. Concurrently, systemic risk factors like Angiotensin II and aldosterone, components of the renin–angiotensin–aldosterone system (RAAS), further exacerbate vascular remodeling through VSMC hypertrophy, ECM remodeling, and fibrosis [113]. Prolonged ischemic stress and metabolic imbalance lead to the activation of programmed cell death pathways in cardiomyocytes and vascular cells. Apoptosis, mediated by mitochondrial dysfunction and caspase activation (e.g., caspase-3, caspase-9), results in the loss of contractile cells and impairs cardiac function [114]. Both pyroptosis and ferroptosis are distinct forms of programmed cell death that contribute to tissue damage in CVD. Pyroptosis promotes vascular inflammation and endothelial injury through caspase-1-dependent activation of gasdermin D, leading to the release of proinflammatory cytokines and cell lysis [115]. On the other hand, ferroptosis is characterized by iron-catalyzed lipid peroxidation and impaired antioxidant defense, primarily due to reduced activity of glutathione peroxidase 4 (GPX4). This process involves iron-catalyzed lipid peroxidation, resulting in membrane disruption and cell death, particularly under conditions of ischemia–reperfusion injury [116]. Experimental data suggest that ferroptosis inhibitors may offer a therapeutic benefit in limiting infarct size and preserving cardiac function [117].

### 4.2. Neurodegenerative Cascades in AD

The amyloidogenic pathway plays a central role in AD pathogenesis and involves the sequential cleavage of the amyloid precursor protein (APP) by β-secretase (BACE1) and γ-secretase, resulting in the formation of Aβ peptides [118]. Aβ42 is particularly prone to aggregation, forming extracellular senile plaques predominantly in the neocortex and hippocampus, which are integral regions for memory and cognition [119]. These plaques disrupt synaptic signaling and initiate neurodegenerative cascades. Soluble Aβ oligomers, considered the most neurotoxic species, interact aberrantly with postsynaptic receptors such as NMDA, AMPA, and cellular prion protein (PrPC), causing calcium homeostasis and exacerbating oxidative stress [120]. These disruptions impair long-term potentiation (LTP), promote long-term depression (LTD), and result in synaptic weakening [121]. In parallel, Aβ aggregates induce robust neuroinflammatory responses by promoting the production of ROS, triggering the release of proinflammatory cytokines (e.g., TNF-α, IL-1β, IL-6), and activating the NLRP3 inflammasome. These processes stimulate microglial-mediated synapse elimination and compromise the integrity of the BBB [122]. This chronic inflammatory state contributes to both synaptic dysfunction and tau hyperphosphorylation by activating kinases, such as glycogen synthase kinase-3β (GSK-3β) and cyclin-dependent kinase 5 (CDK5) [123]. As a consequence, hyperphosphorylated tau dissociates from microtubules and forms neurofibrillary tangles, compromising axonal transport and destabilizing cytoskeletal integrity. These pathological changes, including amyloid and tau abnormalities, can be detected through cerebrospinal fluid (CSF) biomarkers and visualized as brain atrophy using neuroimaging techniques. Together, these tools are valuable for early diagnosis and tracking disease progression.

Synaptic dysfunction arises early in AD and represents a major correlation of cognitive decline. Aβ oligomers preferentially localize to postsynaptic densities, where they perturb receptor composition, scaffold protein organization, and synaptic signaling pathways critical for memory encoding [124]. A crucial molecular mediator of this dysfunction is acetylcholinesterase (AChE), which degrades acetylcholine (ACh) at cholinergic synapses [125]. Degeneration of cholinergic neurons in basal forebrain leads to reduced ACh synthesis, while increased AChE activity at residual synaptic terminals further diminishes ACh availability, thereby worsening memory and attentional impairments [126]. Moreover, AChE has been shown to interact with Aβ peptides, accelerating fibril formation and plaque maturation, suggesting a dual pathological role [127]. Elevated AChE expression has been observed surrounding both senile plaques and neurofibrillary tangles, further amplifying cholinergic deficits and neuronal toxicity [128]. Recent studies also indicate that the complement cascade is activated in response to Aβ, with complement components such as C1q and C3 tagging synapses for microglial-mediated elimination [129,130,131]. This immune activation results in reduced synaptic density, impaired neurotransmission, and ultimately, the loss of functional neural circuits.

Neuroinflammation, driven by activated microglia and astrocytes in response to Aβ and neuronal debris, is increasingly recognized as a key factor in disease progression. Microglia detect Aβ through pattern recognition receptors, TREM2, subsequently activating inflammasome complexes like NLRP3, which promote the release of proinflammatory cytokines including IL-1β, IL-6, and TNF-α [104,132]. Reactive astrocytes further contribute to gliosis and amplify cytokine production. This sustained inflammatory state disrupts synaptic function, inhibits neurogenesis, and damages BBB integrity. Together, these glial-mediated processes intensify Aβ and tau pathology and increase neuronal susceptibility to degeneration.

These inflammatory and pathological cascades ultimately lead to progressive neuronal loss, particularly in the hippocampus and association cortices. Multiple regulated cell death pathways have been implicated in this process [133,134,135]. Intrinsic apoptosis is initiated by an imbalance between pro-apoptotic Bax and anti-apoptotic Bcl-2 proteins, leading to mitochondrial outer membrane permeabilization (MOMP), the release of cytochrome c, and subsequent caspase-3 activation [133]. Pyroptosis contributes to neuroinflammation, mainly via microglial activation through caspase-1 and gasdermin D-mediated pore formation [134]. Ferroptosis, a form of cell death driven by iron accumulation and the depletion of GPX4, leads to lipid peroxidation and synaptic damage in the oxidative environment [135].

### 4.3. The CVD-AD Axis: Shared Pathophysiological Mechanisms

Growing evidence suggests a strong link between CVD and AD, with shared mechanisms such as oxidative stress, vascular dysfunction, inflammation, impaired clearance pathways, etc. [136,137]. The interconnected network between the peripheral cardiovascular system and the central nervous system (CNS) allows for the dynamic transmission of pathology from one to the other. Understanding this interplay is crucial for developing combined treatments that address both diseases simultaneously.

Oxidative stress is one of the earliest and most persistent pathological events in the CVD-AD axis. In CVD, excessive production of ROS by NADPH oxidases and dysfunctional mitochondria impairs NO signaling, which plays a critical role in maintaining vascular homeostasis [136]. This redox imbalance leads to endothelial dysfunction, restricting cerebral perfusion and limiting the delivery of oxygen and nutrients to the brain. The resulting hypoxia triggers the activation of hypoxia-inducible factors (HIFs) and upregulates BACE1, thereby increasing Aβ production [138]. Moreover, hypoxia-induced BBB dysfunction allows peripheral immune cells and inflammatory mediators to enter the CNS. This infiltration promotes neuronal stress, impairs the clearance of misfolded proteins such as Aβ and hyperphosphorylated tau, and ultimately leads to their accumulation [139]. Sustained cerebral hypoperfusion also impairs synaptic transmission and accelerates neuronal injury, contributing to progressive cognitive decline [140].

Redox-driven vascular dysfunction is further exacerbated by chronic hypertension and dyslipidemia, both of which contribute to vascular inflammation and structural degeneration [141]. Persistent hypertension elevates Ang II levels, promoting vasoconstriction and NO depletion, which further restrict blood flow to the brain [142]. In parallel, dyslipidemia promotes foam cell formation and atherosclerotic plaque development, leading to vascular stiffening and narrowed arteries. These structural abnormalities increase leukocyte–endothelial adhesion and facilitate the entry of neurotoxic metabolites into the brain. Once in CNS, these vascular insults activate glial cells and trigger tau phosphorylation via the kinase GSK-3β, thereby impairing synaptic structure and accelerating neurodegenerative cascades [143].

Sustained inflammation links the peripheral vascular system with AD pathology. Circulating cytokines, such as IL-6 and TNF-α, cross the compromised BBB and activate glial cells, initiating a chronic neuroinflammatory cascade [144]. These activated glia cells release secondary ROS and proinflammatory mediators, which contribute to neuronal stress, apoptotic signaling, and tau hyperphosphorylation. Moreover, microvascular damage characterized by pericyte loss and endothelial senescence impairs the crucial link between blood flow and neuronal activity and the glymphatic clearance system of the brain. This disruption leads to a buildup of Aβ in the extracellular space, and the toxic oligomeric forms of Aβ then interfere with normal forms of synaptic plasticity by affecting NMDA and AMPA receptors [145]. In addition, the interplay between Aβ plaques, hyperphosphorylated tau tangles, and activated complement components promotes excessive synaptic pruning by microglia, ultimately causing neuronal death and cognitive decline [122,131,145].

## 5. Multifunctional Preventive Roles of Marine BAPs Targeting the CVD-AD Axis

**Figure 1:**
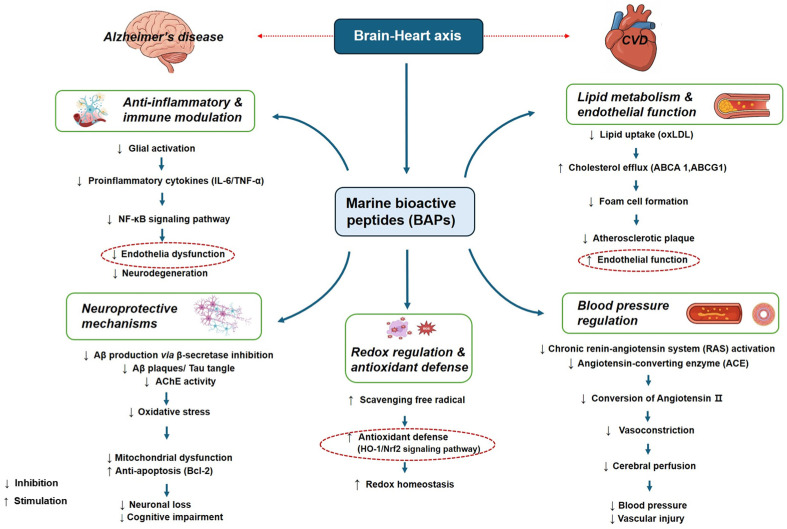
Schematic diagram of marine-derived BAPs in brain–heart axis.

Given the convergence of oxidative stress, inflammation, and vascular dysfunction in CVD and AD, marine BAPs have gained significant attention as multifunctional agents capable of modulating shared pathologies. This section outlines evidence-based roles of marine BAPs in targeting common mechanisms across the CVD-AD axis, structured around core biological processes including oxidative imbalance, inflammation, lipid dysregulation and endothelial impairment, hypertension, and neuronal degeneration. Table 3 summarizes the functional activities of marine-derived BAPs in modulating these pathological processes central to the CVD-AD axis. A schematic diagram of BAPs in the brain–heart axis is demonstrated in Figure 1.

### 5.1. Marine-Derived BAPs in Redox Regulation and Antioxidant Defense

Disrupted redox homeostasis is a shared pathogenic mechanism across CVD and AD, where excessive ROS production impairs endothelial integrity, accelerates lipid peroxidation, and induces neuronal toxicity [146,147,148]. This imbalance not only exacerbates vascular dysfunction but also promotes neurodegeneration through mitochondrial damage, inflammation, and apoptotic signaling [131,132,133,134,135]. Marine-derived BAPs restore redox equilibrium by directly scavenging ROS, inducing antioxidant enzymes, and activating redox-sensitive signaling pathways such as Nrf2/HO-1.

A series of marine peptides exhibit strong direct free radical neutralization activity compared to GSH or vitamin C. Phe-Gly-His-Pro-Tyr from *Mytilus edulis* scavenged hydroxyl radicals with 89.5% efficiency at 64.8 µM, highlighting its potent antioxidative capability [83]. Similarly, Leu-Trp-His-Thr-His from *Styela clava* effectively neutralized peroxyl radicals, contributing to cellular protection [91]. Lys-Ala-Pro-Asp-Pro-Gly-Pro-Gly-Pro-Met from tilapia skin neutralized ROS via hydrogen bonding and hydrophobic interactions [102], while low-molecular-weight collagen hydrolysates from tuna skin exerted ROS-scavenging effects through scavenger receptor-mediated pathways [104]. Additionally, Gly-Val-Ser-Gly-Leu-His-Ile-Asp, derived from *Actinopyga Lecanora*, demonstrated dose-dependent antioxidant activity [75].

Beyond direct ROS neutralization, several peptides enhance endogenous antioxidant defenses. Asn-Cys-Trp-Pro-Phe-Gln-Gly-Val-Pro-Leu-Gly-Phe-Gln-Ala-Pro-Pro from *Marphysa sanguinea* upregulated catalase, SOD, and GPx in LPS-stimulated macrophages, while reducing malondialdehyde (MDA) and proinflammatory cytokines [60]. SWP-I and SWP-II from Atlantic cod swim bladder suppressed β-galactosidase and apoptosis in H_2_O_2_-exposed cells, indicating anti-senescence properties [100]. Peptides from *Piaractus brachypomus*, *Magalaspis cordyla*, and *Otolithes ruber* effectively inhibited lipid peroxidation and ROS accumulation, suggesting broad-spectrum redox mitigation [31,99]. Notably, Gly-Ile-Ile-Gly-Pro-Ser-Gly-Ser-Pro, Ile-Gly-Thr-Gly-Ile-Pro-Gly-Ile-Trp, and Gln-Ile-Gly-Phe-Ile-Trp from *Hippocampus abdominalis* showed dual ACE-inhibitory and alkyl radical scavenging activity, emphasizing their dual benefit in oxidative and vascular modulation [101].

HO-1/Nrf2 axis modulation is another major mechanism through which BAPs exert antioxidative effects. Pro-Ile-Ile-Ser-Val-Tyr-Trp-Lys and Phe-Ser-Val-Val-Pro-Ser-Pro-Lys from blue mussel activated HO-1 via the Nrf2 pathway in HUVECs, providing robust protection against oxidative stress [81]. Similarly, Asn-Gly-Val-Ala-Ala from *Oratosquilla woodmasoni* exhibited both ACE inhibition and antioxidant activity, functioning through direct radical interaction and Nrf2-linked signaling [90].

### 5.2. Blood Pressure Regulation via Inhibiting Renin–Angiotensin System (RAS)

Hypertension is a major pathological contributor to both CVD and AD, acting through impaired endothelial signaling, oxidative stress, and reduced cerebral perfusion [149]. Clinical evidence shows that controlling blood pressure (BP) significantly reduces the risk of cardiovascular problems [150,151]. RAS is a crucial hormonal pathway for regulating BP. Angiotensin-converting enzyme (ACE) plays a central role in this system by converting angiotensin I into the potent vasoconstrictor, angiotensin II. Persistent ACE activity contributes to vascular remodeling and endothelial damage by disrupting NO signaling and promoting apoptotic pathways, ultimately leading to vascular and neurovascular degeneration [152]. Consequently, natural RAS modulators, particularly marine-derived ACE inhibitors, have gained attention for their therapeutic relevance.

Marine algae have yielded potent ACE-inhibitory peptides. For example, Ile-Asp-His-Tyr from *Gracilariopsis chorda* exhibited high ACE binding affinity (−9.50 kcal/mol) via α-helix interactions [56]. Synthetic derivatives Tyr-Ile-Gly-Asn-Asn-Pro-Ala-Lys-Gly-Gly-Leu-Phe and Ile-Gly-Asn-Asn-Pro-Ala-Lys-Gly-Gly-Leu-Phe from *Laminaria digitata* inhibited ACE-1 by over 80% at 1 mg/mL and concurrently suppressed DPP-IV [68]. KAF from *Ulva prolifera* demonstrated strong ACE inhibition (IC_50_: 0.63 µM), with molecular docking showing key hydrogen bonds with Ala354 and Asp415 [71], highlighting their dual regulatory function.

Several mollusk- and echinoderm-derived peptides have demonstrated strong ACE-inhibitory activity, with some also exhibiting endothelial-protective functions. For instance, Gly-Val-Gly-Ser-Pro-Tyr from Pinctada fucata acts as a competitive ACE inhibitor (IC_50_ = 5.82 µg/mL) [86]. Ala-Gly-Phe-Ala-Gly-Asp-Asp-Ala-Pro-Arg and Cys-Asp-Val-Asp-Ile-Arg from Chlamys farreri also showed potent inhibition [87]. Notably, Ile-Lys, Tyr-Glu-Gly-Asp-Pro, and Trp-Phe from Mytilus edulis bind ACE at the catalytic pocket while enhancing NO production and suppressing ROS and endothelin-1 (ET-1) in HUVECs [85]. Asp-Leu-Thr-Asp-Tyr from Crassostrea gigas significantly reduced systolic BP and ACE activity in hypertensive rats [88], and His-Asp-Trp-Trp-Lys-Glu-Arg from Apostichopus japonicus demonstrated similar efficacy via ACE binding and BP reduction in spontaneously hypertensive rats [58].

Ser-Pro and Val-Asp-Arg-Tyr-Phe from *Katsuwonus pelamis* not only exhibited potent ACE inhibitory activity, (IC_50_, 0.06 and 0.28 mg/mL) but also improved endothelial function by promoting NO release and reducing ET-1 expression [95]. Pro-Pro-Leu-Leu-Phe-Ala-Ala-Leu from *Takifugu flavidus* and Val-Ile-Ser-Asp-Glu-Asp-Gly-Val-Thr-His from *Ruditapes philippinarum* displayed combined ACE-inhibitory and antioxidant effects, contributing to endothelial homeostasis [49,61]. Asp-Pro-Ala-Leu-Ala-Thr-Glu-Pro-Asp-Pro-Met-Pro-Phe from *Oreochromis niloticus* enhanced both endothelial and neuronal resilience in BV-2 cells through ACE inhibition and oxidative stress mitigation [96].

Expanding RAS modulation upstream, Ile-Arg-Leu-Ile-Ile-Val-Leu-Met-Pro-Ile-Leu-Met-Ala from *Palmaria palmata* inhibited renin and significantly decreased systolic BP in SHR rats following oral administration at 50 mg/kg [57,70]. This suggests a complementary mechanism beyond ACE targeting, supporting broader intervention strategies. Marine-derived BAPs exhibit multimodal antihypertensive actions by inhibiting ACE and renin, enhancing endothelial resilience, and attenuating oxidative injury. Due to their multi-target effects across the RAS, oxidative stress, and vascular signaling, BAPs are promising candidates for integrated CVD and AD prevention strategies.

In many studies, Captopril^®^ is used as the standard reference for ACE inhibition. Compared with Captopril^®^, the ACE-inhibitory potency of marine-derived BAPs is generally lower, requiring higher peptide concentrations to achieve similar effects. For example, the peptides Tyr-Ile-Gly-Asn-Asn-Pro-Ala-Lys-Gly-Gly-Leu-Phe and Ile-Gly-Asn-Asn-Pro-Ala-Lys-Gly-Gly-Leu-Phe inhibited ACE by over 80% at 1 mg/mL, whereas Captopril^®^ achieved comparable inhibition at a much lower concentration of 0.05 mg/mL [68]. Nevertheless, marine BAPs demonstrate multi-target antihypertensive potential, including enhanced nitric oxide production, reduced endothelin-1 levels, and mitigation of oxidative stress, which likely contribute to their overall vascular and neuroprotective effects.

### 5.3. Regulation of Lipid Metabolism and Endothelial Function

Atherosclerosis underlies both CVD and neurovascular dysfunction in AD, serving as a mechanistic bridge via common processes including lipid accumulation, oxidative stress, and endothelial dysfunction [111]. Chronic exposure to risk factors such as hypertension, dyslipidemia, and hyperglycemia leads to endothelial activation, increased permeability, and foam cell formation, which collectively impair vascular homeostasis and promote systemic and cerebral inflammation [153,154]. In this context, marine BAPs have gained attention as multifunctional modulators capable of restoring lipid balance, reinforcing endothelial stability, and mitigating inflammation-related damage across the CVD-AD axis.

Marine-derived bioactive peptides (BAPs) help regulate lipid homeostasis and foam cell formation, which are early and critical steps in atherogenesis and cognitive decline. For example, blue mussel peptides Pro-Ile-Ile-Ser-Val-Tyr-Trp-Lys and Phe-Ser-Val-Val-Pro-Ser-Pro-Lys significantly reduced lipid-laden foam cell formation in RAW264.7 macrophages and human aortic smooth muscle cells (hASMCs) [79]. Similarly, peptides Leu-Leu-Arg-Leu-Thr-Asp-Leu, Gly-Tyr-Ala-Leu-Pro-Cys-Asp-Cys-Leu, Ala-Trp-Leu-Asn-His, and Pro-His-Asp-Leu from ark shell modulated cholesterol trafficking and exhibited lipid-lowering effects in macrophage models, suggesting that BAPs can correct dyslipidemia-induced inflammation [78,79]. These effects may involve modulation of scavenger receptor activity and restoration of the balance between cholesterol influx and efflux. In these studies, 10 µM of simvastatin and rosiglitazone were used as positive controls.

In parallel, BAPs strengthen endothelial junction integrity and suppress vascular inflammation by modulating adhesion molecules and preserving structural proteins. A conjugate of hydrolyzed jellyfish collagen and black jelly mushroom maintained endothelial viability in EA.hy926 cells under cholesterol stress and increased the expression of VE-cadherin, a key adherents junction protein [85]. An 11-residue peptide derived from *Chlorella* significantly reduced expression of adhesion molecules such as E-selectin, ICAM-1, VCAM-1, and MCP-1 under inflammatory conditions, thereby preserving endothelial barrier function and attenuating leukocyte recruitment, data compared with 0.25 mM of Indomethacin [54].

Through antioxidant defense and anti-apoptotic mechanisms, marine BAPs also confer vascular protection under oxidative stress. Low molecular weight peptides (<1 kDa) from *Mytilus coruscus* reduced oxidative stress and lipid peroxidation by modulating the NF-κB and Nrf2 signaling pathways [84]. His-Gly-Ser-His and Lys-Gly-Pro-Ser-Trp peptides from *Hippocampus abdominalis* activated the HO-1/Nrf2 axis and attenuated endothelial apoptosis [92]. Similarly, Glu-Pro-Thr-Phe and Phe-Thr-Val-Asn peptides derived from blue mussels protected HUVECs from oxidative injury which induced by H_2_O_2_ by enhancing cellular antioxidant defenses and suppressing intrinsic apoptotic pathways [82]. In support of these in vitro observations, marine collagen peptides obtained from wild-caught *Oncorhynchus keta* improved endothelial function in diabetic models [94]. It achieved by reducing both circulating and vascular levels of CF6 and mitochondrial proteins, inhibiting endothelial apoptosis, and increasing PPARγ expression, a nuclear receptor that regulates lipid and glucose metabolism, at doses ranging from 2.25 to 9.0 g/kg/day. These results underscore the multifaceted role of BAPs in maintaining vascular homeostasis under oxidative challenge, supporting their therapeutic relevance to both CVD and AD pathophysiology.

Together, these results demonstrate the multifactorial roles of marine BAPs in regulating lipid metabolism, preserving endothelial integrity, and mitigating oxidative damage. Their coordinated activity across vascular and metabolic axes offers an integrated therapeutic approach for addressing both atherogenesis and neurovascular impairment associated with AD.

### 5.4. Anti-Inflammatory Actions and Immune Modulation by BAPs

Persistent inflammation is a shared pathophysiological hallmark of both CVD and AD, driving vascular injury, immune dysregulation, and neuronal degeneration [155,156,157]. Initiated by endothelial activation, inflammation promotes the expression of adhesion molecules such as ICAM-1 and VCAM-1, facilitating monocyte infiltration and cytokine release, which in turn propagates both atherogenesis and neuroinflammatory damage. The chronic inflammatory environment contributes to BBB dysfunction, ROS overproduction, and neurotoxic cytokine accumulation, serving as a mechanistic bridge between peripheral vascular pathology and central nervous system decline [132].

Marine-derived BAPs have demonstrated significant efficacy in modulating endothelial inflammatory pathways. The octapeptide Ile-Ile-Ala-Val-Glu-Ala-Gly-Cys from *Isochrysis zhanjiangensis* significantly downregulated ICAM-1, VCAM-1, TLR-4, NOS-2, COX-2, IL-6, and TNF-α in LPS-stimulated HUVECs. It also reduced ROS generation by activating the Nrf2/SOD/HO-1 pathway and inhibited both apoptosis and angiogenesis via PI3K/Akt and MAPK signaling [69]. Likewise, Leu-Asp-Ala-Val-Asn-Arg and Met-Met-Leu-Asp-Phe from *Spirulina maxima* suppressed IL-8, MCP-1, and E-selectin expression in histamine-stimulated EA.hy926, operating primarily through PKCδ-regulated MAPK and Egr-1 pathways [53].

Beyond endothelial targets, marine BAPs also attenuate inflammation in immune effector cells. The tripeptide Glu–Thr–Thr from *I. zhanjiangensis* significantly reduced the expression of proinflammatory cytokines (IL-1β, IL-8, TNF-α), inflammatory enzymes (iNOS, COX-2), and vascular signaling components (ET-1, AT-1R) in Ang II-stimulated HUVECs, implicating MAPK/NF-κB/Akt pathways in its mechanism of action. Captopril^®^ (10 mg/kg) was used as the positive control to detect systolic blood pressure [73]. Similarly, collagen-derived peptides Phe-Ala-Gly-Pro-Pro-Gly-Gly-Asp-Gly-Gln-Pro-Gly-Ala-Lys, and Ile-Ala-Gly-Pro-Ala-Gly-Pro-Arg-Gly-Pro-Ser-Gly-Pro-Ala, isolated from *Salmo salar* skin, significantly suppressed NO and proinflammatory cytokine levels in macrophages activated by LPS exposure, indicating potent anti-inflammatory effects within the monocyte-macrophage lineage [97]. Marine fungi are also a source of potent anti-inflammatory compounds. A cyclic tetrapeptide, sclerotiotide-type tripeptide, and diketopiperazine dimer from *Aspergillus violaceofuscus* were found to significantly inhibit IL-10 production in THP-1 cells, with the cyclic tetrapeptide and diketopiperazine dimer achieving 84.3% and 78.1% inhibition, respectively, at concentrations as low as 10 µM [67].

Collectively, these studies underscore the multimodal anti-inflammatory potential of marine-derived BAPs. By targeting both endothelial activation and immune cell cytokine production, BAPs disrupt the sustained inflammatory loops that underlie CVD-AD pathogenesis. Their multifunctional actions across redox, adhesion, and cytokine networks support their integration into strategies aimed at preventing or delaying the progression of both vascular and neurodegenerative diseases.

### 5.5. Integrated Neuroprotective Mechanisms of Marine BAPs

Marine-derived BAPs exert neuroprotective effects by targeting early pathogenic triggers such as antioxidant defense, the anti-amyloidogenic pathway, apoptotic processes, anti-inflammatory regulation, and restoration of cholinergic transmission. Regarding anti-amyloidogenic APP processing, the heptapeptide (Ser-Leu-Ala-Phe-Val-Leu-Asn) and its derivatives inhibited BACE1 by stably binding to the active site of the enzyme, thereby suppressing Aβ production in SH-SY5Y neuroblastoma cells [101]. BAPs also exert anti-neuroinflammatory and apoptotic effects that help preserve neuronal viability. By downregulating the NF-κB-mediated inflammatory enzyme COX-2 and proinflammatory cytokines IL-6 and TNF-α, *Chlorella*-derived peptides attenuated gliosis and neurodegenerative progression in an AD mouse model [55]. The peptide HTP-1 from *Hippocampus trimaculatus* enhanced neuronal survival in Aβ_42_-treated cells by upregulating the anti-apoptotic protein Bcl-2, thereby stabilizing mitochondrial membranes and preventing cytochrome c release [92].

Redox dysregulation, a core driver of neuronal and vascular damage, is also mitigated by marine BAPs. Small peptides (1–10 kDa) derived from *Chlorella pyrenoidosa* protected neuronal cells from glutamate-induced excitotoxic stress by decreasing the accumulation of Aβ and tau. Moreover, these peptides reduced neuronal cell loss and improved spatial cognition and learning memory suggesting that their neuroprotective actions against excitotoxic and Aβ-associated pathological insults may collectively promote cognitive preservation (indomethacin (0.25 mM) as positive control) [54]. In a scopolamine-induced cholinergic dysfunction mouse model, the hexapeptide Phe-Tyr-Asp-Trp-Pro-Lys from sea cucumber enhanced cognitive performance by increasing superoxide dismutase (SOD) activity, reducing MDA levels, and preserving hippocampal neurons, indicating its role in redox stabilization under acute oxidative stress [59].

Marine BAPs have emerged as promising modulators of cholinergic neurotransmission, targeting both enzymatic activity and neuronal homeostasis. Notably, the hexapeptide Leu-Leu-Asp-Phe, characterized from yellowfin tuna, exhibits selective inhibitory activity against AChE, with an IC_50_ value of 8.44 ± 0.24 µM. Molecular docking studies further suggest a stable interaction with the AChE catalytic site via multiple hydrogen bonds, implying a direct role in preserving synaptic acetylcholine levels and stabilizing cholinergic signaling dynamics [42]. By inhibiting AChE activity and restoring mitochondrial function, the sea cucumber-derived heptapeptide Asn-Asp-Glu-Glu-Leu-Asn-Lys elevated acetylcholine levels and reduced ROS accumulation [77]. These effects were linked to the activation of PKA, BDNF, and NGF, suggesting its potential to enhance neuronal resilience under pathological conditions.

## 6. Structure–Activity Relationship (SAR) and Advantages of BAPs in CVD-AD Axis

The biological efficacy of BAPs is primarily dictated by their chemical structures, and structure–activity relationships (SARs) playing a central role in determining functionality [158]. Key structural determinants include amino acid composition and sequence, molecular weight, the nature of N- and C-terminal residues, hydrophobicity/hydrophilicity, and overall charge of the peptide chain. In general, short peptides composed of 2–12 amino acids exhibit enhanced bioactivity, bioavailability, and target specificity, largely due to improved intestinal absorption and greater metabolic stability [159].

Among BAPs relevant to CVD and AD, ACE-inhibitory peptides represent one of the most extensively studied classes of BAPs in CVD and AD prevention. Their antihypertensive activity is strongly dependent on peptide sequence and terminal residues, particularly the C-terminal region. Peptides enriched with hydrophobic, aromatic (Tyr, Trp, Phe), or positively charged residues (Arg, Lys) at the C-terminus display enhanced ACE inhibition, and Pro at the C-terminal further strengthens enzyme binding [160]. In contrast, aliphatic or aromatic residues such as Leu, Ile, and Val at the N-terminus favor antihypertensive efficacy. Although increased C-terminal hydrophobicity is generally associated with stronger ACE inhibition, peptide length, and specific residue identity also play essential roles [161].

Oxidative stress and lipid peroxidation also play central roles in both CVD and neurodegeneration, and antioxidant peptides similarly exhibit distinct SAR patterns, with greater activity reported when Val, Ile, Leu, Ala, Phe, and Lys are positioned at the C-terminus, and when hydrophobic residues (Met, Val, Leu, Pro) are abundant, facilitating lipid solubility and free radical interactions [162]. Additionally, His contributes to metal-ion chelation, and sulfur-containing residues (Met, Cys) enhance redox-based antioxidant mechanisms [158]. Furthermore, it is reported that immunomodulatory peptides are frequently enriched in hydrophobic amino acids such as Gly, Leu, Pro, Phe, and Val, while the presence of Arg at either terminal region plays a critical role in receptor-mediated immune recognition [163].

In the context of AD, neuroprotective peptides consistently contain aromatic and basic residues. Lys and Arg enhance electrostatic interactions and neurotransmitter signaling, whereas Trp, Tyr, and Phe facilitate inhibition of key enzymes such as acetylcholinesterase. Glutamate further contributes through its unique role in brain metabolism and synaptic transmission [164].

Marine-derived peptides offer distinct advantages that complement these SAR-driven effects. Their unique amino acid sequences, shaped by adaptation to extreme environments such as high salinity, variable temperatures, and low oxygen, confer enhanced stability, bioavailability, and multifunctional bioactivity [165]. Structural features, including cyclic conformations and post-translational modifications, improve target selectivity, binding affinity, and resistance to enzymatic degradation, surpassing many linear terrestrial peptides [166]. Marine peptides often exhibit broad-spectrum bioactivities, antihypertensive, antioxidant, immunomodulatory, and neuroprotective, which are particularly relevant to the prevention of CVD and AD. Furthermore, these peptides generally demonstrate low toxicity and high sustainability, making them promising candidates for functional foods, nutraceuticals, and therapeutic applications [167].

Collectively, the biological efficacy of BAPs arises from shared SAR principles, including terminal residue composition, peptide length, hydrophobicity, and charge, while marine-derived peptides offer additional advantages through structural uniqueness, enhanced stability, and multifunctional bioactivity. These combined properties make BAPs, particularly from marine sources, highly promising molecules for health-promoting interventions targeting the CVD-AD axis.

## 7. Current Challenges and Future Perspectives

Despite growing evidence supporting the CVD and AD potential of marine-derived BAPs, several challenges remain that limit their translation into effective functional foods or nutraceuticals targeting the CVD-AD axis. One of the most significant obstacles is the limit in vivo stability and bioavailability of orally administered peptides. Marine BAPs are highly susceptible to degradation by gastrointestinal proteases and rapid systemic metabolism, which markedly reduces their intact absorption and biological efficacy [168]. Following oral ingestion, peptides are exposed to digestive enzymes such as pepsin, trypsin, and chymotrypsin, as well as extensive metabolism by plasma, hepatic, and renal peptidases, leading to substantial losses in stability and bioavailability [169]. Consequently, biological activities observed in vitro are often not fully reproduced in vivo because of extensive gastrointestinal digestion and rapid enzymatic degradation, necessitating high doses or frequent administration and thereby limiting practical applications [170]. Although clinically established peptide therapies typically rely on parenteral or intranasal delivery to overcome these limitations, oral administration remains the most convenient and acceptable route for functional food-based and long-term preventive strategies [171]. Improving resistance to enzymatic degradation and enhancing oral bioavailability therefore remain critical challenges for the effective use of marine BAPs in chronic disease management, including the CVD-AD axis.

Encapsulation and advanced delivery systems have emerged as a promising strategy to overcome these limitations by protecting peptides from both GI degradation and systemic metabolism. Platforms such as liposomes, nanoemulsions, and polymer-based carriers can shield peptides from enzymatic breakdown, control their release, and enhance overall oral bioavailability [172]. Importantly, for neuroprotective effects, the relevance of encapsulation also depends on the ability of peptides to cross the BBB. Lipid-based nanocarriers, including liposomes and solid lipid nanoparticles, can protect peptides from degradation while interacting with BBB endothelial cells, potentially facilitating transcytosis into brain tissue. Functionalization with targeting ligands can further enhance receptor-mediated transport across the BBB via pathways such as low-density lipoprotein or transferrin receptors, or through surface-modified peptides exploiting endogenous transport mechanisms [173]. In addition, intranasal nanoencapsulation provides a non-invasive route that partially bypasses the GI tract and BBB, reducing peptide degradation and improving brain delivery compared with conventional oral or intravenous administration [174]. Overall, receptor-targeted and peptide-functionalized nanoparticles represent a versatile approach to simultaneously address GI stability, systemic bioavailability, and BBB permeability, enhancing the therapeutic potential of marine-derived BAPs for CVD-AD management.

Another important challenge in translating marine-derived BAPs into functional foods or nutraceuticals for CVD-AD prevention is the limited understanding of peptide–drug interactions, particularly in elderly populations. Many marine BAPs share mechanistic targets with commonly prescribed drugs, such as angiotensin-converting enzyme inhibition or modulation of lipid metabolism, raising the possibility of additive or synergistic pharmacodynamic effects, including hypotension or excessive lipid lowering when co-consumed with antihypertensive or statin therapies. For example, peptides derived from *Channa striata* skin collagen and fermented fish products (Bekasam) have demonstrated hypocholesterolemic activity through inhibition of 3-hydroxy-3-methylglutaryl-CoA reductase (HMG-CoAR). In particular, collagenase-hydrolyzed fish skin collagen inhibited HMG-CoAR activity by approximately 25.8% compared with the reference inhibitor pravastatin [175]. In addition, cholesterol-lowering effects have been reported in foam cell models when marine BAPs were co-administered with simvastatin or rosiglitazone, suggesting potential additive or synergistic actions [77,78,79]. While such findings indicate that BAPs may complement existing pharmacotherapies, they also underscore important safety considerations, particularly in the context of age-related changes in drug metabolism, vascular responsiveness, and renal clearance. Future research should therefore prioritize systematic co-administration studies to evaluate safety, pharmacokinetics, and pharmacodynamic interactions, establish dose–response relationships, and define safe intake thresholds. These efforts will be essential for developing evidence-based guidelines and ensuring that marine BAPs are positioned as complementary nutritional interventions rather than unintended pharmacological modifiers in aging populations.

Allergenicity represents an additional challenge for seafood-derived BAPs intended for food applications. Food processing is widely recognized as an effective strategy for mitigating allergenicity by modifying allergen structure and IgE-binding epitopes [176]. Chemical- and biological-based approaches, including enzymatic hydrolysis, cross-linking, and glycation (e.g., Maillard reactions), can induce protein unfolding, peptide chain disruption, aggregation, or selective amino acid modification, thereby masking or eliminating allergenic epitopes and reducing IgE reactivity. Because allergic responses are mediated by specific interactions between IgE antibodies and their cognate epitopes, targeting epitope composition and structure represents a rational mitigation strategy [177]. However, the limited characterization of both linear and conformational epitopes, along with structural rearrangements during processing, may lead to variable or unpredictable allergenic outcomes. Moreover, interactions between allergens and food matrix components can either shield epitopes or promote the formation of novel allergenic structures. Notably, most available evidence is derived from in vitro and animal studies, while clinical validation in seafood-allergic individuals remains limited [178].

A further translational gap exists between experimental findings and industrial implementation, particularly with respect to scalable production, product standardization, and regulatory approval. Although enzymatic hydrolysis of marine proteins provides a cost-effective approach for generating bioactive peptide mixtures, large-scale production of well-defined peptides with consistent and reproducible biological activity remains technically and economically challenging [63]. Collectively, while these approaches remain largely at the experimental stage, they underscore promising directions for improving the delivery and translational potential of marine-derived BAPs. Emerging tools, including computational modeling and bioinformatics-driven optimization, further support peptide discovery and functional characterization. Overall, marine BAPs hold substantial promise as functional food and nutraceutical components, offering integrated preventive strategies that target both cardiovascular disease and Alzheimer’s disease.

## 8. Conclusions

Marine-derived BAPs represent a multifunctional class of bioactive compounds with demonstrated effects relevant to the CVD-AD axis, including antioxidant, anti-inflammatory, antihypertensive, lipid-regulating, and neuroprotective activities. These diverse properties support their potential as preventive agents, particularly through incorporation into functional foods and nutraceuticals. This review integrates current knowledge on functional properties, mechanistic pathways, and sustainable production of marine BAPs, highlighting their value as natural, safe, and eco-friendly resources. Despite these advances, challenges such as scalable production, product standardization, and regulatory approval remain barriers to widespread application. Continued efforts in peptide discovery, characterization, and formulation will be essential to move from preclinical promise to real-world impact. Ultimately, marine BAPs offer a sustainable and innovative avenue for integrated prevention of CVD and AD, aligning with the growing emphasis on functional food-based strategies for chronic disease management. While marine bioactive peptides offer significant therapeutic promise for targeting the cardiovascular–Alzheimer’s disease axis, their sustainable translation will depend on responsible sourcing and the development of scalable biotechnological production methods.

## Figures and Tables

**Table 3:** Functional activities of marine-derived BAPs in the pathophysiology of the CVD-AD axis.

Bioactivity	Peptide (Sequence)	Source	Experimental Model	Mechanism of Action	Ref.
Redox regulation and antioxidant defense	His-Asn-Leu-Gly-Leu-Leu-His-Gly-Asp-Met, Asp-Ala-Pro-Ser-Met-Asn-Asp	*Piaractus brachypomus*	DPPH, ABTS, FRAP	Exhibits strong antioxidant properties.	[31]
	Asn-His-Arg-Tyr-Asp-Dr, Gly-Asn-Arg-Gly-Phe-Ala-Cys-Arg-His-Ala	*Horse mackerel*, *Croaker*	DPPH, hydroxyl radical scavenging	Demonstrates potent free radical scavenging and lipid peroxidation inhibition activities.	[99]
	Gly-Val-Ser-Gly-Leu-His-Ile-Asp	*Actinopyga lecanora*	DPPH, ABTS, FRAP	Exhibits strong radical scavenging capacity.	[75]
	Lys-Ala-Pro-Asp-Pro-Gly-Pro-Gly-Pro-Met	*Oreochromis niloticus*	DPPH, ABTS	Shows significant radical scavenging activity, attributed to hydrogen bonding and hydrophobic interactions with radical species.	[62]
	Low MW peptides (<600 Da)	*Bigeye tuna*	DPPH, ABTS, FRAP	Exerts antioxidant effects through scavenger receptor pathways.	[100]
	GPH-IV-P2	*Perna canaliculus*	DPPH, ABTS	Demonstrates radical scavenging ability.	[89]
	Gly-Ile-Ile-Gly-Pro-Ser-Gly-Ser-Pro, Ile-Gly-Thr-Gly-Ile-Pro-Gly-Ile-Trp, Gln-Ile-Gly-Phe-Ile-Trp	*Hippocampus abdominalis*	Alkyl radical scavenging	Exhibits alkyl radical scavenging activities.	[101]
	Asn-Gly-Val-Ala-Ala	*Oratosquilla woodmasoni*	DPPH, ABTS, super oxide radical scavenging	Displays dual ACE-inhibitory and antioxidant activity.	[90]
	Phe-Leu-Ala-Pro (FLAP)	*Acaudina molpadioides*	DPPH, ABTS	Shows strong DPPH and ABTS radical scavenging activity.	[74]
	Leu-Trp-His-Thr-His	*Styela clava*	Peroxyl radical scavenging	Effectively scavenges peroxyl radicals.	[91]
	Phe-Gly-His-Pro-Tyr	*Mytilus edulis*	Hydroxyl radical scavenging	Exhibits radical scavenging activity.	[83]
	Pro-Ile-Ile-Ser-Val-Tyr-Trp-Lys, Phe-Ser-Val-Val-Pro-Ser-Pro-Lys	*Mytilus edulis*	HUVECs	Activates HO-1/Nrf2 signaling cascade, enhances endogenous antioxidant defense, and reduces pro-apoptotic markers under oxidative stress.	[80]
	Glu-Pro-Thr-Phe, Phe-Thr-Val-Asn	*Mytilus edulis*	HUVECs	Protects HUVECs from H2O2-induced oxidative stress by enhancing the HO-1 antioxidant pathway and downregulating pro-apoptotic markers.	[81]
	<1 kDa peptide fraction	*Mytilus coruscus*	HUVECs	Restores redox homeostasis by reducing ROS/MDA, modulating apoptotic signaling, inhibiting NF-κB, and activating Nrf2 antioxidant pathway.	[84]
	His-Gly-Ser-His, Lys-Gly-Pro-Ser-Trp	*Hippocampus abdominalis*	HUVECs	Reduces apoptosis through inhibition of Bax, cytochrome c, and caspase-3, alongside HO-1/Nrf2 signaling pathway activation.	[94]
	Tyr-Glu-Gly-Asp-Pro, Trp-Phe	*Mytilus edulis*	HUVECs	Protects HUVECs from oxidative stress by enhancing NO production, reducing ET-1 secretion, and increasing antioxidase activities.	[82]
	Asp-Pro-Ala-Leu-Ala-Thr-Glu-Pro-Asp-Pro-Met-Pro-Phe	*Oreochromis niloticus*	BV-2 cells	Displays antioxidant activity by scavenging free radicals.	[96]
	Asn-Cys-Trp-Pro-Phe-Gln-Gly-Val-Pro-Leu-Gly-Phe-Gln-Ala-Pro-Pro	*Marphysa sanguinea*	RAW264.7	Increases the activity of key antioxidant enzymes (CAT, SOD, GSH-Px) and reduces malondialdehyde (MDA) levels.	[60]
	SWP-I, SWP-II	*Gadus morhua*	2BS cells	Exhibits free radical scavenging activity, reduces senescence-associated β-galactosidase (SA-β-gal) activity, and inhibits apoptosis.	[98]
Blood pressure regulation (RAS Inhibition)	Ile-Asp-His-Tyr	*Gracilariopsis chorda*	Human ACE, molecular docking	Strong human ACE inhibition via high binding affinity in molecular docking analyses.	[56]
	Tyr-Ile-Gly-Asn-Asn-Pro-Ala-Lys-Gly-Gly-Leu-Phe, Ile-Gly-Asn-Asn-Pro-Ala-Lys-Gly-Gly-Leu-Phe	*Laminaria digitata*	ACE-1 enzyme, DPP-IV enzyme	Exhibits significant ACE-1 inhibitory activities and shows DPP-IV inhibitory properties.	[68]
	Gly-Val-Gly-Ser-Pro-Tyr	*Pinctada fucata*	ACE	Acts as a potent competitive inhibitor of ACE.	[86]
	Lys-Ala-Phe	*Ulva prolifera*	ACE, molecular docking	Strong ACE inhibition, stability of KAF-ACE complex is mediated by hydrogen bonds with critical residues (Ala354, Asp415).	[71]
	Ile-Lys, Tyr-Glu-Gly-Asp-Pro, Trp-Phe, Ser-Trp-Ile-Ser-Ser	*Mytilus edulis*	ACE, HUVECs	Interacts strongly with ACE active site via hydrogen bonds, electrostatic forces, and hydrophobic contacts, YEGDP and WF enhance NO production and antioxidase activities.	[83]
	Asp-Asp-Gln-Ile-His-Ile-Phe, His-Asp-Trp-Trp-Lys-Glu-Arg, Thr-His-Asp-Trp-Trp-Lys-Glu-Arg	*Apostichopus japonicus*	ACE, SHRs	Inhibits ACE by deep embedding in its binding pocket, stabilizing the complex. In vivo, HDWWKER significantly lowers systolic BP.	[58]
	Ser-Pro, Val-Asp-Arg-Tyr-Phe	*Katsuwonus pelamis*	ACE	Binds effectively to ACE via hydrogen bonding, electrostatic, and hydrophobic interactions, enhances NO production and decreases ET-1 secretion.	[95]
	Pro-Pro-Leu-Leu-Phe-Ala-Ala-Leu	*Takifugu flavidus*	ACE, SHRs	Inhibits ACE via multiple hydrogen bonds, hydrophobic contacts, and coordination with the ACE zinc ion. In vivo, reduces BP.	[61]
	Ala-Gly-Phe-Ala-Gly-Asp-Asp-Ala-Pro-Arg, Cys-Asp-Val-Asp-Ile-Arg (and others)	*Chlamys farreri*	ACE	Contains N-terminal Ile, Ala, Met, Leu, basic Arg, Lys, His, and C-terminal aromatic Tyr, Phe, Trp residues, associated with potent ACE inhibition.	[87]
	Asp-Leu-Thr-Asp-Tyr	*Crassostrea gigas*	SHRs	Significantly reduces systolic BP and ACE activity in SHRs in vivo.	[88]
	Met-Glu-Gly-Ala-Gln-Glu-Ala-Gln-Gly-Asp	*Acaudina molpadioidea*	SHRs	Shows strong antihypertensive effects in SHRs, implying ACE inhibition.	[73]
	Asp-Pro-Ala-Leu-Ala-Thr-Glu-Pro-Asp-Pro-Met-Pro-Phe	*Oreochromis niloticus*	BV-2 cells	Exhibits potent ACE inhibition and antioxidant activity by scavenging free radicals.	[96]
	Ile-Arg-Leu-Ile-Ile-Val-Leu-Met-Pro-Ile-Leu-Met-Ala	*Palmaria palmata*	Renin enzyme, SHRs	Inhibits renin. In vivo, leads to a significant reduction in systolic BP.	[57,70]
	GPH-IV-P2	*Perna canaliculus*	ACE	Demonstrates radical scavenging ability and inhibits ACE activity.	[89]
	Gly-Ile-Ile-Gly-Pro-Ser-Gly-Ser-Pro, Ile-Gly-Thr-Gly-Ile-Pro-Gly-Ile-Trp, Gln-Ile-Gly-Phe-Ile-Trp	*Hippocampus abdominalis*	ACE	Potent ACE inhibitory activity through hydrogen bonding and active site interactions, also exhibits antioxidant properties.	[101]
	Asn-Gly-Val-Ala-Ala	*Oratosquilla woodmasoni*	ACE	Displays dual ACE-inhibitory and antioxidant activity.	[90]
Regulation of lipid metabolism and endothelial function	Pro-Ile-Ile-Ser-Val-Tyr-Trp-Lys, Phe-Ser-Val-Val-Pro-Ser-Pro-Lys	*Mytilus edulis*	RAW264.7 macrophages, hASMCs	Inhibits foam cell formation and suppresses lipid accumulation via cholesterol efflux pathways.	[79]
	Leu-Leu-Arg-Leu-Thr-Asp-Leu, Gly-Tyr-Ala-Leu-Pro-Cys-Asp-Cys-Leu, Ala-Trp-Leu-Asn-His, Pro-His-Asp-Leu	*Scapharca subcrenata*	RAW264.7 macrophages	Reduces intracellular lipid accumulation and modulates cholesterol metabolism.	[77,78]
	Hydrolyzed collagen	*L. smithi* + black jelly mushroom extract	EA.hy926 cells	Preserves viability, reduces apoptosis, enhances VE-cadherin expression, improving endothelial junction integrity.	[85]
	Val-Glu-Cys-Tyr-Gly-Pro-Asn-Arg-Pro-Gln-Phe	*Chlorella pyrenoidosa*	RAW264.7 macrophages and SVEC4-10 cells co-culture	Suppresses adhesion molecules (E-selectin, ICAM-1, VCAM-1) and MCP-1 expression while maintaining endothelial barrier function.	[54]
	Marine collagen peptides (MCPs)	Chum salmon skin	HUVECs, diabetic Wistar rats	Improves endothelial function by decreasing circulating/arterial CF6 and MP, inhibits endothelial apoptosis and increases PPARγ expression.	[93]
	Glu-Pro-Thr-Phe, Phe-Thr-Val-Asn	*Mytilus edulis*	HUVECs	Protects against H2O2-induced oxidative stress by enhancing the HO-1 antioxidant pathway and downregulating pro-apoptotic markers.	[81]
	<1 kDa peptide fraction	*Mytilus coruscus*	HUVECs	Restores redox homeostasis by reducing ROS/MDA, modulating apoptotic signaling, inhibiting NF-κB, and activating Nrf2 antioxidant pathway.	[84]
	His-Gly—Ser-His, Lys-Gly-Pro-Ser-Trp	*Hippocampus abdominalis*	HUVECs	Reduces apoptosis by inhibiting Bax, cytochrome c, and caspase-3, alongside HO-1/Nrf2 pathway activation.	[94]
Anti-Inflammatory actions and immune modulation by BAPs	Pro-Ile-Ile-Ser-Val-Tyr-Trp-Lys, Phe-Ser-Val-Val-Pro-Ser-Pro-Lys	*Mytilus edulis*	RAW264.7 macrophages	Suppresses NF-κB pathway activation, reducing expression of proinflammatory cytokines and iNOS/COX-2.	[79]
	Leu-Leu-Arg-Leu-Thr-Asp-Leu, Gly-Tyr-Ala-Leu-Pro-Cys-Asp-Cys-Leu, Ala-Trp-Leu-Asn-His, Pro-His-Asp-Leu	*Scapharca subcrenata*	RAW264.7 macrophages	Suppresses proinflammatory cytokines (e.g., TNF-α, IL-6) and reduces NO production via NF-κB signaling inhibition	[77,78]
	Leu-Asp-Ala-Val-Asn-Arg, Met-Met-Leu-Asp-Phe	*Spirulina maxima*	RBL-2H3 mast cells	Reduces IL-8, IL-6, MCP-1, adhesion molecules, and ROS by downregulating Egr-1 via histamine receptor- and PKCδ-dependent MAPK pathways.	[53]
	Glu-Thr-Thr	*Isochrysis zhanjiangensis*	HUVECs	Decreases proinflammatory cytokines (IL-1β, IL-8, TNF-α), iNOS, COX-2, ET-1, and AT-1 levels by inhibiting MAPK, NF-κB, and Akt signaling.	[72]
	Ile-Ile-Ala-Val-Glu-Ala-Gly-Cys	*Isochrysis zhanjiangensis*	HUVECs	Downregulates inflammatory mediators (ICAM-1, VCAM-1, TLR-4, NOS-2, COX-2, IL-6, TNF-α), inhibits ROS via Nrf2/SOD/HO-1 and NF-κB pathways.	[71]
	Phe-Ala-Gly-Pro-Pro-Gly-Gly-Asp-Gly-Gln-Pro-Gly-Ala-Lys, Ile-Ala-Gly-Pro-Ala-Gly-Pro-Arg-Gly-Pro-Ser-Gly-Pro-Ala	*Salmo salar*	RAW264.7 macrophages	Strongly suppresses the production of key inflammatory mediators (NO, IL-6, IL-1β, TNF-α) in LPS-stimulated RAW264.7 macrophages.	[97]
	Cyclic tetrapeptide, Aspochracin-type cyclic tripeptide, Sclerotiotide L, Diketopiperazine dimer	*Aspergillus violaceofuscus*	THP-1 cells	Suppresses IL-10 production in LPS-induced THP-1 cells.	[67]
	Asn-Cys-Trp-Pro-Phe-Gln-Gly-Val-Pro-Leu-Gly-Phe-Gln-Ala-Pro-Pro	*Marphysa sanguinea*	RAW264.7 macrophages	Downregulates NO, iNOS, COX-2, IL-1β, and TNF-α.	[60]
Integrated neuroprotective mechanisms of marine BAPs	Leu-Leu-Asp-Phe	Yellowfin tuna	AChE enzyme, molecular docking	Exhibits notable AChE inhibitory activity by strongly binding to its active site via hydrogen bonding.	[42]
	CPPs (<1 kDa to >10 kDa fractions)	*Chlorella pyrenoidosa*	Neuronal cells, AD mouse model	Enhances neuronal viability, reduces Aβ plaques and tau tangles, and reduces neuroinflammation via downregulation of IL-6, TNF-α, COX-2, and NF-κB. In vivo, improves cognitive function.	[55]
	Asn-Asp-Glu-Glu-Leu-Asn-Lys	*Stichopus japonicus*	PC12 cells	Elevates acetylcholine, reduces AChE activity, enhances SOD/lowers ROS (antioxidant), suppresses mitochondrial dysfunction, acts via PKA/BDNF/NGF pathway.	[76]
	HTP-1	*Hippocampus trimaculatus*	PC12 cells	Protects PC12 cells from Aβ_42_-induced neuronal death by upregulating anti-apoptotic gene Bcl-2, inhibiting apoptosis.	[92]
	Ser-Leu-Ala-Phe-Val-Leu-Asn	*Merluccius productus*	β-secretase enzyme, SH-SY5Y cells	Potent β-secretase inhibitory activity confirmed by high binding affinity in molecular docking, neuroprotective in SH-SY5Y cells.	[102]
	Phe-Tyr-Asp-Trp-Pro-Lys	*Stichopus japonicas*	Male Kunming mice	Counters scopolamine-induced cognitive impairment by enhancing SOD, reducing MDA, and reducing neuronal loss, acts via antioxidant, anti-cholinergic, and anti-inflammatory pathways.	[59]

## Data Availability

No new data were created or analyzed in this study. Data sharing is not applicable to this article.

## Abbreviations

The following abbreviations are used in this manuscript:

CVD	Cardiovascular diseases
AD	Alzheimer’s diseases
BAPs	Bio-active peptides
PUFAs	Linear dichroism
HPLC	High-performance liquid chromatography
FPLC	Fast protein liquid chromatography
SPPS	Solid-phase peptide synthesis
QSAR	Quantitative structure-activity relationship
Bead-GPS	Bead-based global proteomic screening
NO	Nitric oxide
ROS	Reactive oxygen species
LDL	Low-density lipoproteins
oxLDL	Oxidized low-density lipoproteins
VCAM-1	Adhesion molecule-1
ICAM-1	Intercellular adhesion molecule-1
VSMCs	Vascular smooth muscle cells
RAAS	Renin–angiotensin–aldosterone system
GPX4	Glutathione peroxidase 4
BACE1	Amyloid precursor protein (APP) by β-secretase
PrPC	Prion protein
LTP	Impair long-term potentiation
LTD	Promote long-term depression
GSK-3β	Glycogen synthase kinase-3β
CDK5	Cyclin-dependent kinase 5
MDA	Malondialdehyde
AChE	Acetylcholinesterase
ACh	Acetylcholine
MOMP	Mitochondrial outer membrane permeabilization
CNS	Central nervous system
HIFs	Hypoxia-inducible factors
ACE	Angiotensin-converting enzyme
hASMCs	Human aortic smooth muscle cells
BBB	Blood–brain barrier
